# m^6^A modification of a 3′ UTR site reduces *RME1* mRNA levels to promote meiosis

**DOI:** 10.1038/s41467-019-11232-7

**Published:** 2019-07-30

**Authors:** G. Guy Bushkin, David Pincus, Jeffrey T. Morgan, Kris Richardson, Caroline Lewis, Sze Ham Chan, David P. Bartel, Gerald R. Fink

**Affiliations:** 1 Whitehead Institute for Biomedical Research, Cambridge, MA 02142 USA; 2 Department of Molecular Genetics and Cell Biology and the Center for Physics of Evolving Systems, University of Chicago, Chicago, IL 60637 USA; 3 Department of Biology, Massachusetts Institute of Technology, Cambridge, MA 02139 USA; 4 Howard Hughes Medical Institute, Cambridge, MA 02142 USA

**Keywords:** Gene regulation, RNA modification, Meiosis, Enzymes

## Abstract

Despite the vast number of modification sites mapped within mRNAs, known examples of consequential mRNA modifications remain rare. Here, we provide multiple lines of evidence to show that Ime4p, an *N*6-methyladenosine (m^6^A) methyltransferase required for meiosis in yeast, acts by methylating a site in the 3′ UTR of the mRNA encoding Rme1p, a transcriptional repressor of meiosis. Consistent with this mechanism, genetic analyses reveal that *IME4* functions upstream of *RME1*. Transcriptome-wide, *RME1* is the primary message that displays both increased methylation and reduced expression in an Ime4p-dependent manner. In yeast strains for which *IME4* is dispensable for meiosis, a natural polymorphism in the *RME1* promoter reduces *RME1* transcription, obviating the requirement for methylation. Mutation of a single m^6^A site in the *RME1* 3′ UTR increases Rme1p repressor production and reduces meiotic efficiency. These results reveal the molecular and physiological consequences of a modification in the 3′ UTR of an mRNA.

## Abstract (web summary)

Ime4p is a yeast N6-methyladenosine (m6A) methyltransferase with an unknown role in meiosis. Rme1p is a repressor of meiosis. Here the authors show that Ime4p methylates *RME1* 3′ UTR to reduce its expression and enable meiosis, thus providing an example of an m6A site with a physiological role.

## Introduction

N^6^-methyladenosine (m^6^A), one of the hundreds of posttranscriptional RNA modifications known to occur in RNA^1^, is the most common internal modification in eukaryotic mRNA^2,3^. The mRNA methyltransferase is a protein complex in which one of its members, encoded by the *IME4* gene in yeast (Initiator of MEiosis 4), harbors the catalytic activity^4^. In many yeast strains, *IME4* is required for progression through meiosis^5,6^, and the recognition that it encodes an mRNA methyltransferase has provided the initial evidence that internal mRNA modifications have physiological consequences^4^.

Ime4p is conserved among eukaryotes, with characterized homologs in mammals (METTL3)^7^, Drosophila (dIME4)^8^, and Arabidopsis (MTA)^9^. Other noncatalytic members of the yeast methyltransferase complex include Slz1p and Mum2p^10,11^. *MUM2* also has homologs in mammals (WTAP)^12,13^, Drosophila (Fl(2)d)^14^, and Arabidopsis (FIP37)^9,15^. The m^6^A mark is found in a conserved consensus motif within mRNAs^16,17^ and thought to be deposited co-transcriptionally based on its presence in chromatin-associated pre-mRNA exons^18^, co-localization of dIme4 and PolII on Drosophila chromosomes^19^, and increased m^6^A levels in transcripts with reduced transcription rates^20^. m^6^A is most often found in proximity to stop codons, in either the coding sequence or the 3′ untranslated region (UTR)^21–23^, and has been broadly linked to diverse aspects of mRNA metabolism and function, including altered splicing^19,24–26^, decreased mRNA stability^27–29^, and altered translational efficiency^20,28,30–32^.

Some of the consequences of m^6^A mRNA methylation rely on “reader” proteins, which have a YTH domain that interacts specifically with m^6^A^33–37^. YTHDF1 and YTHDF3 bind to a subset of m^6^A mRNAs and enhance their translation, and in the case of YTHDF1 this increased translation efficiency results from its interaction with members of the eIF3 complex^26,38^. YTHDF2 is implicated in mRNA degradation, as its knockdown results in increased mRNA half-life of m^6^A mRNAs^29^. Deletion of YTHDF2 in mice results in female infertility^39^. Similarly, YTHDC2 deletion in mice results in infertility due to meiotic arrest during gametogenesis^40^. In Drosophila, dIME4 is expressed in gonads and required for gametogenesis^8^. When considered together with the *ime4*-*Δ* phenotype in yeast, these results suggest that m^6^A has an evolutionarily conserved role in meiosis.

Meiosis in *S. cerevisiae* is part of the sporulation-differentiation program, in which four haploid spores are formed from one diploid cell^41^. The decision to enter meiosis is controlled through multiple pathways that integrate nutritional and ploidy signals. Diploid cells enter meiosis when starved for nitrogen in the presence of a non-fermentable carbon source. The master regulator of meiosis in yeast is a transcriptional activator called Initiator of meiosis 1 (Ime1p). *IME1* has one of the longest promoters in the yeast genome, which harbors binding sites for proteins that transmit the nutritional status signals^42^ and two binding sites for Rme1p (Regulator of meiosis 1), a DNA-binding protein that prevents meiosis in haploids by repressing *IME1* transcription^43–46^. In diploids, *RME1* is itself repressed by the product of the mating-type alleles, the a1/α2 complex^44^. However, genetic and mutational analyses suggest that there are additional unidentified mechanisms of *RME1* repression^44^. *IME4*, which encodes the mRNA m^6^A methyltransferase in yeast, is known to be required for *IME1* expression, but how this positive regulation is achieved has been unclear^6^. Furthermore, it is unclear why *IME4* is necessary for meiosis in some yeast strains^6^ but dispensable in others^10^.

The regulatory impact of m^6^A modifications within mRNAs, inferred from phenotypic and molecular analyses of mutations that disrupt m^6^A writers or readers in yeast, plants, flies, and mammals, is now broadly appreciated^47–49^. Less progress has been made in distinguishing the consequential m^6^A sites out of the entire m^6^A methylome. Thus far, one functional m^6^A modification has been reported in an intron of the mammalian *S-adenosylmethionine (SAM) synthase* mRNA, which regulates its splicing^25,50^. Two functional m^6^A sites were reported in the 5′ UTRs of mammalian *HSP70* and *ATF4*, where they regulate cap-independent translation and alternative translation, respectively^51,52^. However, the vast majority of mapped m^6^A sites are near the stop codon and at the 3′ UTR^21,22^, for which there are no known physiologically relevant examples validated by mutational analysis. In this study, we identify a consequential m^6^A site within the *RME1* 3′ UTR that reduces *RME1* mRNA levels to enable meiotic progression. Thus *IME4* lies upstream of *RME1* in the meiotic entry pathway, which explains the decades-old observation that *IME4* is required for *IME1* expression. These results reveal the molecular consequences of a modification in the 3′ UTR of an mRNA and explain the strain-dependent requirement for methylation of mRNA.

## Results

### A promoter polymorphism reduces *RME1* expression

**Fig. 1:**
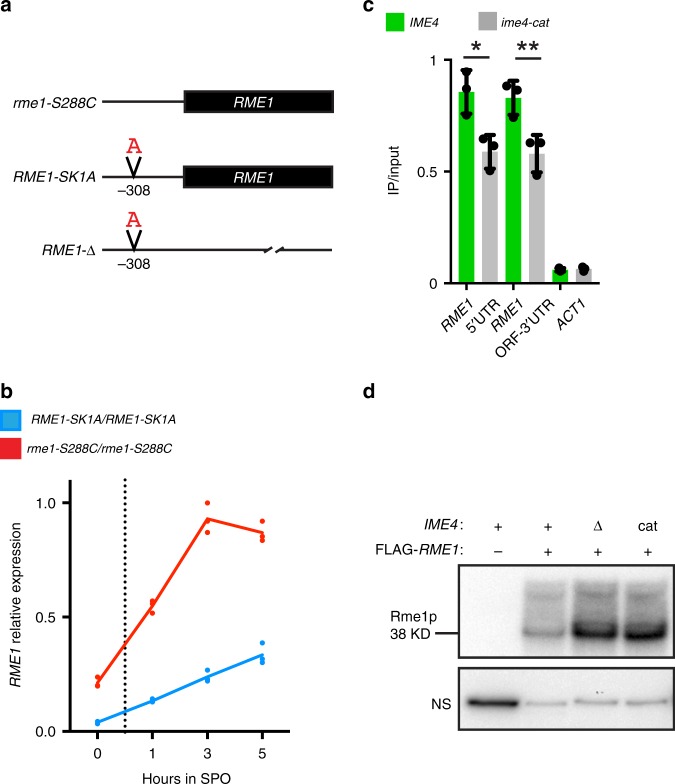
*RME1* alleles in this study. *IME4* downregulates Rme1p through its m^6^A activity. **a** The three *RME1* alleles are in the SK288C strain background. *RME1-SK1A* has a single A insertion 308 nucleotide upstream of the open reading frame (ORF) relative to *rme1-S288C*. **b** Quantitative reverse transcription PCR (RT-qPCR) quantification of *RME1-SK1A* and *rme1-S288C* expression during meiosis. Means and individual values from three biological replicates. Source data are provided in a Source Data file. **c** m^6^A immunoprecipitation (IP) followed by RT-qPCR using *RME1* 5′ untranslated region (UTR) primers or primers that span the end of the ORF and the 3′ UTR on mRNA isolated from *IME4* and *ime4-cat* homozygous cells, both in *rme1-S288C* homozygous cells. Transcript levels were first normalized to *PGK1* as an internal control, and then the fold enrichment of IP/input was calculated. The residual enrichment of *RME1* transcripts in *ime4-cat*/*ime4-cat* cells relative to *IME4*/*IME4* cells probably results from non-specific binding to the anti-m^6^A antibody^13,23^. *ACT1* serves as a non-methylated control. Means, individual values, and s.d. from three biological replicates. *p = 0.022, **p = 0.019, two-tailed *t* test. Source data are provided in a Source Data file. **d** Western blot using anti-FLAG antibody showing Rme1p expression from the *rme1-S288C* allele in strains homozygous for the indicated allelic backgrounds incubated in SPO media for 5 h. A non-specific (NS) band present in all lanes serves as a loading control. Source data are provided in a Source Data file

Polymorphisms in several genes account for most of the difference in meiotic efficiency between two interbreeding strains of *Saccharomyces cerevisiae*: SK1 (high efficiency) and S288C (low efficiency)^53,54^. An S288C strain that has three alleles replaced with SK1 alleles—named SK288C—approaches SK1 in its elevated meiotic efficiency^54,55^. The SK1 allele with the largest contribution to meiotic efficiency is *RME1-SK1*, which differs in sequence from that of *rme1-S288C* by three nucleotides^54^. One of these polymorphisms, insertion of an A (ins-308A, Fig. 1a) located in the upstream non-coding region of *RME1*, is solely responsible for efficient meiotic sporulation^54^. We named this allele *RME1-SK1A* and the allele in S288C *rme1-S288C*. Reverse transcription followed by quantitative PCR (RT-qPCR) showed that *RME1-SK1A* expression is reduced nearly four-fold compared with the *rme1-S288C* allele (Fig. 1b), which is the allele present in most sequenced laboratory strains (Supplementary Fig. 1a). Because Rme1p is a DNA-binding protein that directly represses the meiotic transcriptional program, this reduced transcription of *RME1* explains the increase in meiotic efficiency in SK1. Our following studies on *IME4* function utilize the SK288C strain, containing the highly expressed *rme1-S288C*, the poorly expressed *RME1-SK1* allele, or a deletion of the entire *RME1*-coding region (*RME1-Δ*).

### *IME4* represses Rme1p expression

SK1 and S288C differ because *IME4* is essential for meiosis in S288C but dispensable for meiosis in SK1 (Supplementary Fig. 1b^10^). *IME4* encodes a methyltransferase that directs the posttranscriptional conversion of A to m^6^A in mRNA^4^. To determine whether the differential *IME4* dependence is due to a role for Ime4p and/or m^6^A in regulating *RME1* expression, we generated a strain homozygous for a deletion of *IME4* (*ime4-Δ*/*ime4-Δ*) and a strain homozygous for a catalytically inactive allele (*ime4-cat*/*ime4-cat*)^4^. The Ime4p and ime4p-cat proteins were expressed to the same levels during meiosis and vegetative growth (Supplementary Fig. 1c). m^6^A immunoprecipitation (m^6^A IP) of mRNA purified from meiotic *IME4*/*IME4* and *ime4-cat*/*ime4-cat* cells captured less *RME1* mRNA from *ime4-cat*/*ime4-cat* lysate, suggesting that *RME1* transcripts carried the m^6^A modification (Fig. 1c).

Does methylation of *RME1* mRNA affect the expression of Rme1p? We found that both *ime4-Δ*/*ime4-Δ* and *ime4-cat*/*ime4-cat* cells had increased Rme1p levels relative to *IME4*/*IME4* (Fig. 1d and Supplementary Fig. 1d). IP confirmed the increase in Rme1p in these cells (Supplementary Fig. 1e). Rme1p appeared to be posttranslationally modified in meiosis to produce higher-molecular-weight species (Supplementary Fig. 1d). These higher-molecular-weight forms of Rme1p were dithiothreitol (DTT)-resistant, migrate too fast to be sodium dodecyl sulfate (SDS)-resistant homo-oligomers, and did not react with anti-ubiquitin antibodies (Supplementary Fig. 1e). Thus Ime4p is required both for the methylation of *RME1* mRNA and for reduced expression of Rme1p during meiosis.

### Ime4p reduces *RME1* mRNA levels

**Fig. 2:**
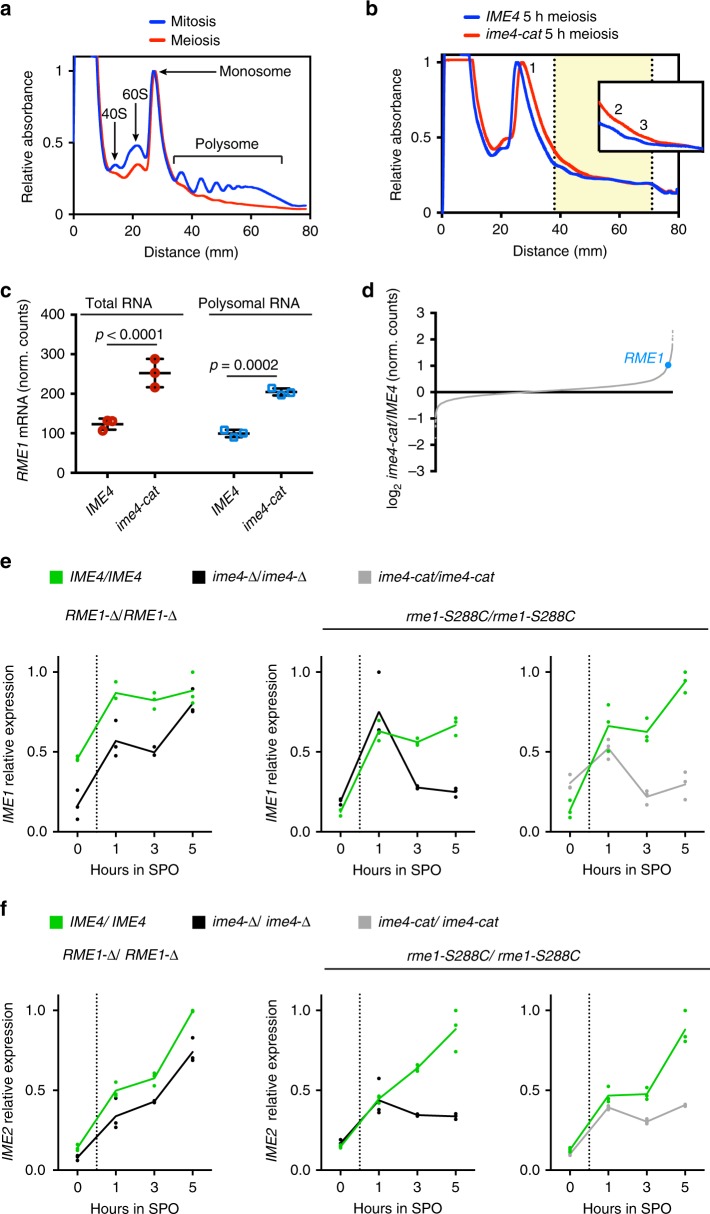
*IME4* m^6^A activity reduces *RME1* mRNA levels to enable meiotic initiation. **a** Polysome profiles (absorbance at 254 nm vs. distance from the top of the tube) of mitotic cells during logarithmic growth and 2 h into meiosis. The locations of the 40S, 60S, monosome, and polysome peaks are indicated. Source data are provided in a Source Data file. **b** Polysome profiles of meiotic *IME4*/*IME4* and *ime4-cat*/*ime4-cat* cells, both in *rme1-S288C*/*rme-S288C*. The inset is a magnification of the polysome area with the number of ribosomes in each peak. The highlighted area marks the pooled fractions used for RNA-seq of polysome-associated mRNA. Source data are provided in a Source Data file. **c** RNA-seq quantifications of *RME1* transcript from input mRNA prior to gradient fractionation (total mRNA) and pooled polysome fractions (polysomal RNA) from *IME4*/*IME4* and *ime4-cat*/*ime4-cat* meiotic cells. Individual values, means, and s.d. from three biological replicates. Two-way analysis of variance *p* values are indicated. Source data are provided in a Source Data file. **d** Rank order plot of mRNA levels in total RNA from *ime4-cat*/*ime4-cat* and *IME4*/*IME4* meiotic cells. Means from three biological replicates. Source data are provided in a Source Data file. **e** Left: Quantitative reverse transcription PCR (RT-qPCR) quantification of *IME1* expression in *RME1-Δ*/*RME1-Δ* during meiosis in *IME4*/*IME4* and *ime4-Δ*/*ime4-Δ* cells. Middle: RT-qPCR quantification of *IME1* expression in *rme1-S288C*/*rme1-S288C* during meiosis in *IME4*/*IME4* and *ime4-Δ*/*ime4-Δ* cells. Right: RT-qPCR quantification of *IME1* expression in *rme1-S288C*/*rme1-S288C* during meiosis in *IME4*/*IME4* and *ime4-cat*/*ime4-cat* cells. Means and individual values from three biological replicates. Source data are provided in a Source Data file. **f** As in **e** above but with measurements of *IME2* expression. Means and individual values from three biological replicates. Source data are provided in a Source Data file

To reduce Rme1p levels, Ime4p catalytic activity might alter either mRNA levels or translational efficiency. To distinguish between these possibilities, we separated the actively translating pool of mRNA from total mRNA by sucrose-gradient polysome fractionation of *IME4*/*IME4* and *ime4-cat*/*ime4-cat* meiotic lysates. Compared to mitotic cells, meiotic cells had a marked reduction in polysomes (Fig. 2a)^56^. Nonetheless, *IME4*/*IME4* and *ime4-cat*/*ime4-cat* cells exhibited comparable meiotic polysome profiles (Fig. 2b), indicating that loss of m^6^A did not affect the ribosomal landscape. RNA-seq of the input and polysome fraction revealed that the total levels of *RME1* mRNA were increased in *ime4-cat*/*ime4-cat* cells compared to *IME4*/*IME4* cells and that there was no further increase in polysome-associated *RME1* mRNA (Fig. 2c). Therefore, the increase in Rme1p can be explained by the increase in *RME1* mRNA. Parallel analyses of other mRNAs showed that such an increase in mRNA was unusual and not observed for >98% of the other mRNAs (Fig. 2d). Taken together, these data indicate that Ime4p catalytic activity specifically decreases Rme1p production by reducing transcript abundance rather than polysome association.

### *RME1* repression enables the meiotic transcriptional program

Rme1p blocks meiosis by preventing activation of the *IME1* gene, which encodes the master transcriptional activator of meiosis^42,57–59^. *IME1* is necessary for meiotic DNA replication^57,60^, even in the presence of the *RME1-SK1A* allele (Supplementary Fig. 1f). To test whether reduced *RME1* mRNA de-represses *IME1*, the level of *IME1* mRNA was determined by RT-qPCR in *IME4*/*IME4*, *ime4-Δ*/*ime4-Δ*, and *ime4-cat*/*ime4-cat* cells. As expected, in the *RME1-Δ*/*RME1-Δ* background, we observed no difference in *IME1* mRNA levels between *IME4*/*IME4* and *ime4-Δ*/*ime4-Δ* cells after 5 h in SPO media (Fig. 2e). By contrast, in the *rme1-S288C*/*rme1-S288C* background, *IME1* levels were reduced more than three-fold in both *ime4-Δ*/*ime4-Δ* and *ime4-cat*/*ime4-cat* cells relative to *IME4*/*IME4* cells (Fig. 2e). Analysis of the time courses showed that an initial burst of *IME1* expression was induced equally in *IME4*/*IME4*, *ime4-Δ*/*ime4-Δ*, and *ime4-cat*/*ime4-cat* cells, but *ime4-Δ*/*ime4-Δ* and *ime4-cat*/*ime4-cat* cells failed to sustain elevated *IME1* expression. This role of Ime4p in sustaining high-level *IME1* expression by overcoming Rme1p repression coincided with the timing of its induced expression (Supplementary Fig. 1c). We observed an increase in *IME1* mRNA levels in early time points in *IME4*/*IME4* compared to *ime4-Δ*/*ime4-Δ* cells in the *RME1-Δ*/*RME1-Δ* background. This increase suggests that Ime4p has an additional role in *IME1* regulation independent of *RME1* (Fig. 2e), although this role was not further investigated. Perhaps Ime4p is involved in transduction of the nutritional starvation signals required to induce *IME1*^42,61^. One of the target genes induced by Ime1p is *IME2*, which encodes a protein kinase required for the expression of many other meiotic genes^58,59,62^. *IME2* expression in the different *RME1* backgrounds paralleled that of *IME1*, indicating that the effects of Ime4p regulation of *RME1* propagate through *IME1* induction to activate the downstream meiotic targets (Fig. 2f).

### A conditional *IME4* requirement for meiotic DNA replication

**Fig. 3:**
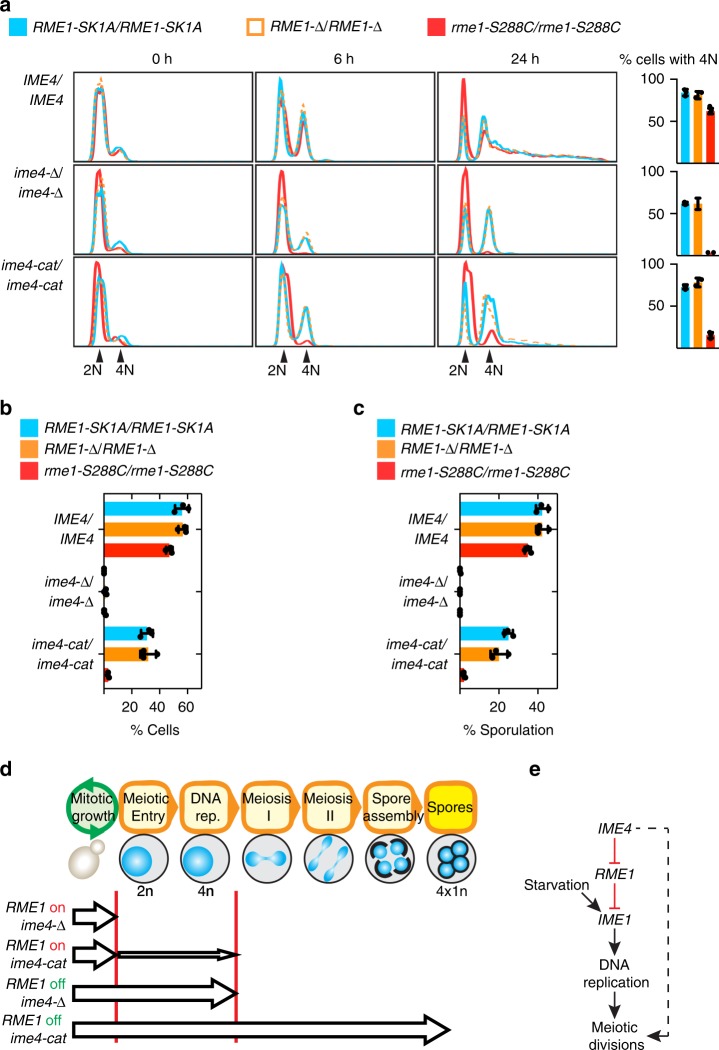
*IME4* m^6^A is required for meiotic DNA replication, and a non-m^6^A function is required for meiotic divisions. **a** Flow cytometric analysis of DNA content in *IME4* and *ime4* mutants (rows) in three color-coded *RME1* allele backgrounds over a meiotic time course (columns). A quantification of the 24-h time point is on the right of each row. Percentage of cells with 4*N* includes both sporulated and non-sporulated cells. Means, individual values, and s.d. from two to five experiments. Source data are provided in a Source Data file. **b** Meiotic nuclear divisions in *IME4* and *ime4* mutants in three color-coded *RME1* allele backgrounds as assayed by DAPI staining of nuclei after 24 h in SPO medium. The percentage of cells with two or more nuclei is indicated on the horizontal axis. Means, individual values, and s.d. from three experiments. At least 200 cells were counted per strain per experiment. Source data are provided in a Source Data file. **c** Sporulation after 48 h in SPO media in *IME4* and *ime4* mutants in three color-coded *RME1* allele backgrounds as scored by light microscopy. Means, individual values, and s.d. from three experiments. At least 200 cells were counted per strain per experiment. Source data are provided in a Source Data file. **d** A model depicting the various points along the *S. cerevisiae* meiotic program in which different *IME4* functions are needed in different *RME1* backgrounds. “*RME1* on” denotes the highly expressed *rme1-S288C* allele, while “*RME1* off” denotes either the hypomorph *RME1-SK1A* or *RME1-Δ*. Arrows represent an ability to progress through the various landmarks that appear at the top, while vertical red lines indicate an inability to progress further. **e** A model for the functions of *IME4* in meiosis: *IME4* promotes meiotic DNA replication by repression of *RME1*, which represses the master activator of meiosis, *IME1*. *IME1* is also activated by nutritional signals. *IME4* has a second function downstream of *IME1* in promoting meiotic divisions (dashed line)

The transcriptional program induced in meiosis initially consists of genes required to carry out meiotic DNA replication. As a functional readout of this process, we measured DNA content by flow cytometry in *IME4*/*IME4*, *ime4-Δ*/*ime4-Δ*, and *ime4-cat*/*ime4-cat* cells in the *RME1-Δ*, *RME1-SK1A*, and *rme1-S288C* homozygous backgrounds following 6 and 24 h in SPO media (Fig. 3a). Deletion of *IME4* led to delayed meiotic DNA replication in cells with no or low *RME1* expression (*RME1-Δ* and *RME1-SK1A*, respectively). However, in the presence of high *RME1* expression (*rme1-S288C*), we detected no DNA replication in *ime4-Δ*/*ime4-Δ* cells, even after 24 h. Cell sorting followed by microscopy revealed that cells to the right of the 4*N* peak at 24 h were asci (Supplementary Fig. 2a, b). The DNA-replication defect observed in *ime4-Δ*/*ime4-Δ* cells was not quite as severe in *ime4-cat*/*ime4-cat* cells. Although DNA replication was substantially delayed in *ime4-cat*/*ime4-cat* cells in the *rme1-S288C* background, some cells ultimately were able to replicate their DNA. Thus the *ime4-cat* allele was incompletely penetrant, suggesting that Ime4p might perform both methyltransferase-dependent and -independent functions.

If *IME4* and *RME1* were in the same pathway, then the DNA-replication phenotypes of the single- and double-deletion strains would indicate their order in the pathway and whether they activate or inhibit each other (Supplementary Table 3 lists the predicted phenotypes for each of the eight possible models). For this analysis, a double-deletion strain of *RME1* and *IME4* was constructed, and meiotic DNA synthesis was measured and compared with that of the wild-type *RME1* and *IME4* strain and the respective single-deletion strains (Fig. 3a). Only the double-repression model, in which *IME4* acts upstream to repress *RME1*, which in turn represses DNA replication, explained all the single- and double-deletion experimental data (Supplementary Table 3). *IME4* is known to be required for the expression of *IME1*^6^. Our results show that this positive genetic relationship is achieved via negative regulation of *RME1* (Fig. 3e).

### *RME1* dosage tightly controls meiotic DNA replication

Our observation that the ~3–4-fold reduction in *RME1* expression caused by the *RME1-SK1A* allele (as compared to *rme1-S288C*, Fig. 1b) had a dramatic effect on meiotic efficiency suggests that the downstream events in meiosis are sensitive to *RME1* dosage. To examine the effects of *RME1* transcript levels on meiotic progression, we analyzed DNA synthesis in various strains. Heterozygotes containing half the amount of *rme1-S288C* (*rme1-S288C*/*RME1-Δ*) replicated their DNA earlier than *rme1-S288C* homozygotes (Supplementary Fig. 3a). Furthermore, in the absence of *IME4* (*ime4-Δ*/*ime4-Δ*), *rme1-S288C*/*RME1-Δ* cells replicated their DNA, whereas *rme1-S288C*/*rme1-S288C* did not. These data demonstrate that a two-fold reduction in *RME1* levels is sufficient to bypass the meiotic defects of a deletion of *IME4*. Moreover, heterozygotes containing one copy of the poorly expressed *RME1-SK1A* allele and one copy of the highly expressed *rme1-S288C* allele (*ime4-Δ*/*ime4-Δ RME1-SK1A*/*rme1-S288C*) proceeded through DNA replication, whereas *ime4-Δ*/*ime4-Δ rme1-S288C*/*rme1-S288C* homozygotes did not (Supplementary Fig. 3b). Consistent with these results, in strains homozygous for the *ime4-cat* allele, *rme1-S288C*/*RME1-Δ* and *RME1-SK1A*/*rme1-S288C* heterozygotes replicated their DNA earlier than *rme1-S288C* homozygotes.

### A methyltransferase activity-independent Ime4p function

We monitored segregation of DNA into distinct nuclei by microscopy and found that, in *ime4-Δ*/*ime4-Δ* cells, no meiotic divisions occurred in any *RME1* allelic background (Fig. 3b and Supplementary Fig. 3c). This indicates that *IME4* is required downstream of DNA replication for the onset of Meiosis I. However, Ime4p catalytic activity was not required for DNA segregation: meiotic divisions occurred efficiently in *ime4-cat*/*ime4-cat* cells, provided they were able to replicate their DNA (*RME1-Δ or RME1-SK1A* backgrounds). Analysis of sporulation efficiency showed that it precisely mirrored DNA segregation in the *ime4* mutants in the various *RME1* backgrounds (Fig. 3c and Supplementary Fig. 3c). We conclude that, in the presence of an active *RME1* allele, *ime4-Δ*/*ime4-Δ* cells do not replicate their DNA, whereas *ime4-cat*/*ime4-cat* cells are severely defective in DNA replication and those that do replicate their DNA arrest following DNA replication (Fig. 3d). In the absence of an active *RME1* allele, *ime4-Δ*/*ime4-Δ* cells arrest following DNA replication, whereas *ime4-cat*/*ime4-cat* cells are able to complete meiosis and sporulate. Together these data reveal that Ime4p functions twice during meiosis: once as an m^6^A methyltransferase that promotes DNA replication, and again before Meiosis I in a catalysis-independent manner to promote DNA segregation (Fig. 3e).

### *RME1* mRNA is an m^6^A target

**Fig. 4:**
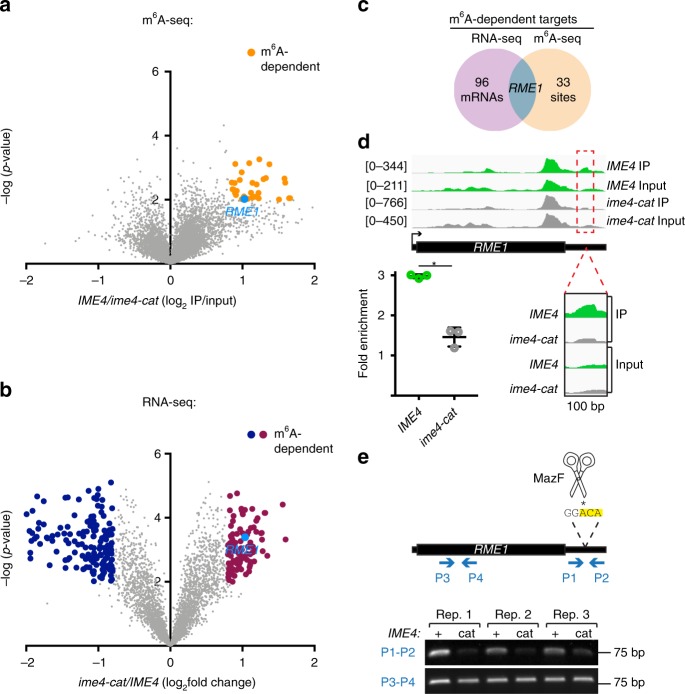
*RME1* mRNA harbors an m^6^A site at its 3′ untranslated region (UTR). **a** m^6^A-seq data from meiotic *IME4* homozygotes and *ime4-cat* homozygotes. Data from three biological replicates are presented as immunoprecipitation (IP) to input ratio in *IME4*, divided by the same ratio in *ime4-cat*. m^6^A sites enriched >1.75-fold in *IME4* relative to *ime4-cat* cells that have a *p* value of <0.01 in a two-tailed *t* test are highlighted in orange. **b** RNA-seq data from meiotic *IME4* homozygotes and *ime4-cat* homozygotes cells. Data from three biological replicates are presented as normalized counts in *ime4-cat*, divided by normalized counts in *IME4*. mRNAs increased >1.75-fold in *ime4-cat*/*ime4-cat* relative to *IME4*/*IME4* cells (*p* value <0.01, two-tailed *t* test) are highlighted in purple. mRNAs decreased >1.75-fold in *ime4-cat*/*ime4-cat* relative to *IME4*/*IME4* cells (*p* value < 0.01, two-tailed *t* test) are highlighted in blue. **c** Venn diagram showing the overlap between the m^6^A-seq data and the RNA-seq data. **d** *RME1* transcript with its 5′ and 3′ UTRs. IGV genome browser views of reads enriched following IP with anti-m^6^A antibodies in *IME4*/*IME4* and *ime4-cat*/*ime4-cat* cells are shown for the entire transcript (top, autoscale read counts on the left of each track), and in a 100-bp window of the 3′ UTR (bottom, reads in this window were normalized to input controls). Fold enrichment of IP/input for reads in the window are shown on the left with means and s.d. from three biological replicates. *p = 0.0006, two-tailed *t* test. Source data are provided in a Source Data file. **e** PCR amplification of cDNA prepared from MazF-digested meiotic mRNA from *IME4*/*IME4* and *ime4-cat*/*ime4-cat* cells. Three biological replicates. P1 and P2 primer locations relative to the probed methylation site are indicated. The MazF ACA site within the methylation site is highlighted in yellow, and the probed nucleotide is marked with an asterisk. Primers P3 and P4, which do not flank an ACA site, were used as control. Source data are provided in a Source Data file

To identify Ime4p methylation targets critical for progression of the meiotic program, we performed m^6^A IP followed by RNA-seq (m^6^A-seq)^21,22^ on RNA from the 5-h meiotic time point of *rme1-S288C*/*rme1-S288C* cells, in which Ime4p catalytic activity is necessary for meiosis. This m^6^A-seq experiment contrasts with previous analyses performed in the SK1 background, where Ime4p m^6^A activity is dispensable for meiosis^23^. We identified 118 sites on 117 transcripts enriched in *IME4*/*IME4* cells relative to *ime4-cat*/*ime4-cat* cells (Supplementary Data 1, see “Methods”). Using these data, we reconstructed the known m^6^A methylation consensus motif and reproduced the observation that m^6^A sites are enriched near the 3′ ends of mRNAs (Supplementary Fig. 4a–c)^21–23^. Of the 117 methylated mRNAs we identified, 51 were also identified in the previous m^6^A-seq dataset from the meiotic-efficient SK1 (Supplementary Fig. 4d, *p* < 0.0001, Fisher’s Exact Test^23^). Analysis leveraging our biological replicates revealed 34 high-confidence m^6^A sites on 34 different mRNAs in *IME4*/*IME4* cells compared with *ime4-cat*/*ime4-cat* cells (log_2_ fold change >0.8, *p* < 0.01, two-tailed *t* test) (Fig. 4a and Supplementary Data 1). The set of high-confidence m^6^A targets included *RME1*.

In a separate experiment using *rme1-S288C*/*rme1-S288C* cells at the 5-h meiotic time point, we also measured the global mRNA levels of *IME4*/*IME4* and *ime4-cat*/*ime4-cat* cells. Analysis of these RNA-seq data revealed 97 mRNAs with significantly elevated levels (log_2_ fold change >0.8, *p* < 0.01, two-tailed *t* test) in *ime4-cat*/*ime4-cat* cells relative to *IME4*/*IME4* cells (Fig. 4b and Supplementary Data 2). These 97 transcripts were enriched for ribosome- and amino acid synthesis-related gene ontologies (GOs; Supplementary Fig. 5a). Parallel analysis of the 156 mRNAs elevated in *IME4*/*IME4* cells relative to *ime4-cat*/*ime4-cat* cells revealed enrichment for meiosis-related categories including synapsis, recombination, homologous chromosome segregation, and sister-chromatid segregation (Supplementary Fig. 5b). Remarkably, the intersection of the set of 97 messages elevated in the absence of Ime4p methyltransferase activity with the set of 34 high-confidence m^6^A targets contained one gene: *RME1* (Fig. 4c).

### Mutation of *RME1* 3′ UTR m^6^A site increases its mRNA levels

A parsimonious explanation for our results so far is that Ime4p directly methylates *RME1* mRNA at a specific site, and the m^6^A mark reduces *RME1* mRNA stability. Analysis of the m^6^A-seq data indeed revealed a single significant peak in the *RME1* message located in the 3′ UTR that was enriched three-fold in *IME4*/*IME4* cells relative to *ime4-cat*/*ime4-cat* cells (Fig. 4d). This peak is centered on a putative m^6^A site 129 nt downstream of the stop codon (+129A), which matched the consensus m^6^A motif (ANRG-m^6^A-CNNU). In order to probe the methylation status of +129A, we used MazF, a methylation-sensitive RNA restriction enzyme^63^, in a recently described PCR-based assay^64^. MazF cleaves RNA at ACA sites but not at m^6^ACA sites. The ACA sequence is part of the GGACA sequence flanking +129A (Fig. 4e). We digested purified mRNA from *IME4*/*IME4* and *ime4-cat*/*ime4-cat* cells with MazF and then reverse transcribed with random hexamers. PCR amplification of the resulting cDNA using primers that flank +129A in the *RME1* 3′ UTR yielded a product in cDNA prepared from *IME4*/*IME4* but not from *ime4-cat*/*ime4-cat* (Fig. 4e). Thus +129A in the *RME1* 3′ UTR is protected from cleavage by MazF in mRNA derived from cells with a functional methyltransferase. This result confirms that the *RME1* 3′ UTR is methylated at the +129A position in *IME4*/*IME4* cells but not in *ime4-cat*/*ime4-cat* cells.

**Fig. 5:**
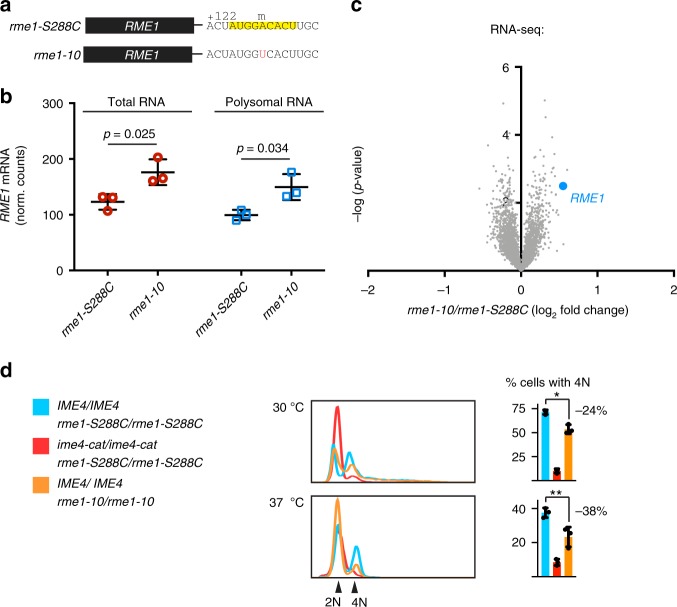
*RME1* methylation mutants are defective in meiotic DNA replication. **a** *rme1-S288C* and *rme1-10* have the same promoter and coding sequence, but they differ in their 3′ untranslated region: *rme1-10* has a single A to T substitution of the +129 methylated A (marked with an m over it) in the methylation consensus motif (yellow). **b** RNA-seq quantifications of *RME1* mRNA. *RME1* mRNA from input RNA prior to gradient fractionation (total mRNA) and polysome-associated RNA (polysomal RNA) from *IME4*/*IME4 rme1-S288C*/*rme1-S288C* and *IME4*/*IME4 rme1-10*/*rme1-10* cells incubated in SPO media for 5 h. Individual values, means, and s.d. from three biological replicates. Two-way analysis of variance *p* values are indicated. Because this experiment was performed together with the one shown in Fig. 2c, the results for *RME1* mRNA in the *IME4* background in Fig. 2c are re-plotted in this panel for the *rme1-S288C* background. Source data are provided in a Source Data file. **c** RNA-seq data from *rme1-S288C* homozygotes and *rme1-10* homozygotes incubated for 5 h in SPO media. Data from three biological replicates are presented as normalized counts in *rme1-10*, divided by normalized counts in *rme1-S288C*. **d** Flow cytometric analysis of DNA content over a meiotic time course in *IME4*/*IME4 rme1-10*/*rme1-10* compared to *IME4*/*IME4 rme1-S288C*/*rme1-S288C* and *ime4-cat*/*ime4-cat rme1-S288C*/*rme1-S288C*. Cells were incubated for 24 h at 30 °C or 37 °C, as indicated. Means, individual values, and s.d. from three biological replicates. *p = 0.011, **p = 0.034, two-tailed *t* test. Source data are provided in a Source Data file

To determine whether methylation of this +129A was important for *RME1* regulation, we mutated the A to a T in the *RME1* genomic locus (Fig. 5a, *rme1-10*). RNA-seq of *rme1-10*/*rme1-10* cells showed a significant increase in total *RME1* mRNA levels and polysomal mRNA levels relative to *rme1-S288C*/*rme1-S288C* (Fig. 5b). Transcriptome-wide analysis of mRNAs in *rme1-10*/*rme1-10* and *rme1-S288C*/*rme1-S288C* meiotic cells revealed that *RME1* was among the most differentially expressed genes (1.46-fold induction, *p* = 0.003, two-tailed *t* test, Fig. 5c). Comparison of *rme1-10*/*rme1-10* and *ime4-cat*/*ime4-cat* cells revealed a significant correlation in mRNA expression levels across the transcriptome (Supplementary Fig. 5c). When compared to wild-type cells (*IME4*/*IME4 rme1-S288C*/*rme1-S288C*), these mutants shared an increase in mRNAs belonging to ribosome- and amino acid synthesis-related ontologies and a decrease in mRNAs belonging to nucleosome- and replication fork-related ontologies (Supplementary Fig. 5d, e)—hallmarks of proliferating cells.

### *RME1* 3′ UTR m^6^A regulates meiotic DNA replication

As expected from increased Rme1p production, *IME4*/*IME4 rme1-10*/*rme1-10* cells were defective in DNA replication compared to *IME4*/*IME4 rme1-S288C*/*rme1-S288C* cells (Fig. 5d). The DNA-replication defect was less severe than that of *ime4-cat*/*ime4-cat S288C*/*rme1-S288C* cells, suggesting that the m^6^A activity of Ime4p also acts on other targets (or other sites on *RME1*) to promote meiotic DNA synthesis. Nonetheless, the single-nucleotide substitution in the m^6^A site of *rme1-10* was sufficient to reduce meiotic DNA replication by 24%, accounting for nearly one third of the replication defect due to complete loss of m^6^A in *ime4-cat*/*ime4-cat* cells. The effect of *rme1-10* was even greater at 37 °C, a restrictive temperature that decreases meiotic efficiency: *me1-10*/*rme1-10* cells had a 38% reduction in DNA replication compared to *IME4*/*IME4* cells, accounting for half of the replication defect in *ime4-cat*/*ime4-cat* cells. Thus this single m^6^A site within *RME1* is required to dampen Rme1p production and enable efficient meiosis.

## Discussion

The presence of the *RME1-S288C* allele in most laboratory strains including S288C, Sigma127b, W303, and RM11 (Supplementary Fig. 1a) suggests that these strains have been selected for a strict mitosis–meiosis dichotomy. The highly sporulating SK1 is an outlier among laboratory strains as it carries the hypomorphic *RME1-SK1A* allele, which has a promoter mutation that reduces transcription of the *RME1* repressor. Our data show that the requirement for *IME4* and its catalytic methyltransferase function for meiosis is dependent upon the particular allele of *RME1* carried by a strain. The SK288C strain is an excellent host for this analysis because it can be assessed for meiotic proficiency either with the fully functional *rme1-S288C* allele or with the hypomorphic *RME1-SK1* allele. In a strain carrying the *rme1-S288C* allele, Ime4p and its catalytic methyltransferase function are required for efficient meiosis: meiotic DNA replication is abolished in *ime4-Δ*/*ime4-Δ* cells and severely reduced in *ime4-cat*/*ime4-cat* cells (Fig. 3a). By contrast, meiotic DNA replication proceeds effectively in a strain carrying the *RME1-SK1A* allele without *IME4* function (*ime4-Δ*/*ime4-Δ*), albeit at a slightly reduced rate. These results show that meiotic mRNA methylation is required only when *RME1* is highly expressed; *IME4* function is necessary to overcome repression of the meiotic program enforced by high levels of *RME1* mRNA. Moreover, a small difference in *RME1* transcription (two-fold) has a dramatic effect on meiotic DNA synthesis, implying that meiotic progression is very sensitive to the levels of *RME1* (Supplementary Fig. 3a, b).

Ime4p m^6^A methyltransferase activity is needed to reduce expression of *RME1* mRNA (Fig. 2c). This downregulation is important because expression of *RME1* mRNA continues throughout meiosis (Fig. 1b)^65^. Given the posttranscriptional nature of the m^6^A modification, the lower mRNA expression level is presumably mediated by the destabilization of modified *RME1* mRNA. Rme1p represses *IME1*, the transcriptional activator of meiosis; consequently, downregulation of *RME1* enables meiotic entry by relieving repression of *IME1* and permitting subsequent DNA replication. By contrast, in strains in which *RME1* is deleted or in strains that harbor the poorly expressed *RME1-SK1A* allele, m^6^A is dispensable for *IME1* expression and subsequent DNA replication (Figs. 2e and 3a). This observation positions *IME4* as an upstream inhibitor of *RME1* repression of *IME1* (Fig. 3e). This upstream placement explains previous observations that *IME4* is required for *IME1* induction in some strains but not in the SK1 strain^6,10^.

This model in which *IME4* functions upstream of *RME1* for meiotic initiation is supported by epistasis analysis of DNA replication in the single and double *IME4* and *RME1* deletion strains. If *IME4* and *RME1* are in the same pathway, only one of the eight possible models is consistent with experimental data: *IME4* represses *RME1*, which represses DNA replication (Fig. 3a and Supplementary Table 3). Although *ime4-Δ*/*ime4-Δ* cells cannot replicate their DNA in the *RME1-S288C* background at any detectable level, *ime4-cat*/*ime4-cat* cells show only a ~75% reduction in DNA replication. Perhaps the effect of Ime4p on its target mRNAs is partially mediated through its interaction with the methylated transcript, in which case, m^6^A might function to stabilize this protein–RNA interaction. In this scenario, Ime4p-cat might still be able to bind its target mRNA with reduced affinity in the absence of its m^6^A catalytic activity, which would explain its intermediate phenotype compared to *ime4-Δ*.

Our results show that the *RME1* 3′ UTR harbors a functional m^6^A site at +129A (Fig. 4d, e): the *rme1-10* mRNA that lacks the m^6^A site has significantly increased mRNA levels compared to wild-type *RME1* mRNA (Fig. 5b). As a result, *IME4*/*IME4 rme1-10*/*rme1-10* cells have reduced DNA replication compared to *IME4*/*IME4 rme1-S288C*/*rme1-S288C* cells. The single-nucleotide substitution in the *RME1* 3′ UTR accounts for ~1/3 to ~1/2 of the defect in *ime4-cat*/*ime4-cat* cells, validating the relevance of this m^6^A site to the meiotic program (Supplementary Fig. 5c and Fig. 5d). The increase of *RME1* mRNA levels in *IME4*/*IME4 rme1-10*/*rme1-10* cells suggests a destabilizing effect for m^6^A on *RME1* transcripts. This is consistent with a role for m^6^A in mRNA destabilization in mammals mediated by the reader protein YTHDF2^29^, which has a homolog in yeast *MRB1*^23^.

Previous studies have used anti-m^6^A IP or anti-m^6^A crosslink IP followed by RNA-seq (m^6^A-seq and miCLIP, respectively) to map thousands of m^6^A sites in the transcriptomes of various cell types in numerous eukaryotes in which m^6^A plays developmental roles^13,15,21–24^. Despite this wealth of m^6^A data and the global effects of perturbing m^6^A deposition and recognition, little is known about the physiological relevance of individual m^6^A sites. However, at least three specific regulatory sites have been validated by mutational analysis: (1) An intronic m^6^A causes intron retention and rapid degradation of the SAM synthase mRNA when SAM levels are high;^25,50^ (2) in the 5′ UTR of mammalian *ATF4* mRNA, m^6^A directs upstream-open reading frame (ORF)-mediated alternative translation during amino acid starvation;^52^ (3) in the 5′ UTR of mammalian *HSP70* mRNA, m^6^A enables cap-independent translation during heat shock^51^. Thus yeast *RME1* provides the founding example of a consequential modification site within an mRNA 3′ UTR.

## Methods

### Strains and sporulation

Strain genotypes are shown in (Supplementary Table 1). Unless otherwise noted, all strains were constructed in the SK288C background^54,55^. To induce meiosis and sporulation, cells were grown in YPD (1% yeast extract, 2% peptone) supplemented with 4% glucose for 25 h at 30 °C with shaking and diluted to OD600 = 0.2 in BYTA media^66^ (1% yeast extract, 2% tryptone, 1% potassium acetate, 50 mM potassium phthalate) and grown for an additional 16.5 h at 30 °C with shaking. Next, cells were washed once with water, re-suspended in SPO media (0.3% potassium acetate) to OD600 = 2, and incubated at 30 °C with shaking.

### *RME1* phylogenetic analysis

Sequence of the *RME1* ORF and 500 nt upstream region in different strains of *S. cerevisiae* were obtained from aligned, assembled genomes from the Sanger Institute’s Saccharomyces Genome Resequencing project (https://www.sanger.ac.uk/research/projects/genomeinformatics/sgrp.html) using the alicat.pl tool, and the Sigma 1278b genome^67^. A maximum likelihood phylogenetic tree was generated with dnaml^68^ and visualized with ClustalX^69^.

### Quantitative reverse transcription PCR

RNA was extracted as follows: Frozen cells (~24 OD600) were disrupted by vortexing in 600 μl AE buffer (50 mM sodium acetate, 10 mM EDTA, 1% SDS) and 600 μl acid phenol (Fisher Scientific) in the presence of ~100 μl acid-washed glass beads (Sigma) at 4 °C for 5 min. RNA was extracted by incubation in phenol at 65 °C for 10 min. Next, cells were vortexed again as before, incubated at 65 °C for 10 min, vortexed, and spun down at 18,400 × *g* for 10 min at 4 °C. The aqueous top phase was transferred to a new tube and extracted again in phenol. After another spin down and transfer to a new tube, RNA was extracted in 400 μl chloroform followed by ethanol precipitation. cDNA was made with SuperScript III (Life Technologies) using random hexamers or gene-specific primers from 1 μg of total RNA. RT-pPCR was performed using SYBR green PCR master mix (Life Technologies) with primers listed in Supplementary Table 2 on Applied Biosystem 7500 or QuantStudio 5 instruments.

### Western blotting

Ten OD600 of 2 OD/ml meiotic cells or cells grown to ~1 OD600/ml in YPD were collected by centrifugation, re-suspended in 1 ml water, spun down briefly, re-suspended in 5% w/v trichloroacetic acid, and incubated on ice for at least 10 min. Cells were then spun down at 18,400 × *g* for 2 min, and the supernatant was aspirated. The pellet was then washed in 500 μl non-pH-ed Tris to adjust the pH. After another centrifugation of cells and aspiration of the supernatant, the pellet was re-suspended in 140 μl of water. Next, 20 μl of 1 M DTT and 40 μl of 5× SDS loading buffer were added. Samples were then incubated for 5 min in a boiling water bath, put on ice, and vortexed just before gel electrophoresis using BioRad Criterion 10% Tris-HCl precast gels at 100 V for ~5–10 min and then at 120 V for ~90 min in Tris glycine SDS buffer. The gel was washed twice in water to remove SDS and equilibrated in transfer buffer (Tris glycine buffer with 15% methanol). Proteins were then transferred onto PVDF membranes (0.45-μM pore, Millipore) pre-incubated in methanol overnight at 15 V at 4 °C. Next, membranes were blocked in 5% milk in TBST (TBS buffer with 0.1% Tween 20) for 1 h at room temperature with shaking. Next, membranes were blotted with the following antibodies at the following dilutions in 5% milk TBST for 1 h at room temperature with shaking: M2 anti-FLAG-HRP (1:25,000, Sigma A8592-2MG), anti-Pgk1-HRP (1:500,000, Abcam 22C5D8), 10F3 anti-HA-HRP (1:10,000, Roche 12 013 819 001), and P4D1 anti-Ub-HRP (1:10,000, Enzo BML-PW0935-0025). Membranes were then briefly washed twice in TBST, followed by 5 more washes for 5 min each with shaking. Next, membranes were developed with supersignal West femto (Life Sciences) for 5 min before imaging with BioRad ChemiDoc XRS+ imaging system. For quantifications, a dilution series from each sample was first run to test signal linear range for each antibody. Proteins levels were then normalized to Pgk1p loading control using the Image J software. Uncropped and unprocessed blot images are in the Source Data file.

### Protein IP

Cells from meiotic cultures incubated for 5 h in SPO were harvested by vacuum filtration and flash frozen in liquid nitrogen. Pellets were resuspended in 1 ml IP lysis buffer (50 mM HEPES pH 8.0, 150 mM NaCl, 1% Triton X-100, 0.1% deoxycholate, 5 mM EDTA, 1× cOmplete Mini EDTA-free protease inhibitor) and lysed by vortexing twice for 5 min with 200 μl acid-washed glass beads (Sigma) at 4 °C. Lysates were then cleared by centrifugation in a microcentrifuge at 21,100 × *g* for 10 min at 4 °C. Supernatants were transferred to new tubes and 40 μl were kept aside as input. In all, 25 μl per sample of M2 anti-FLAG magnetic beads (Sigma M8823) were washed twice in 200 μl IP lysis buffer and then added to each protein lysate sample followed by an overnight incubation on a rotating rack at 4 °C. The next morning, IP samples were washed 3 times in 900 μl ice-cold IP lysis buffer with vortexing and 5 min incubation on ice after each wash. Next, FLAG-Rme1p was eluted using 100 μg/ml FLAG peptide (Sigma) in lysis buffer. Three 100-μl elutions were done per sample and pooled. Eluted proteins were then precipitated by addition of 4 volumes of acetone pre-chilled overnight to −20 °C. Samples were incubated at −20 °C for 1 h and centrifuged at 15,000 × *g* for 10 min at 4 °C. Pellets were then washed in 1 ml chilled acetone and incubated at −20 °C for 1 h followed by another centrifugation as before. Next, protein pellets were dried in a biosafety hood, taking care not to over dry the pellets, and resuspended in 40 μl of purified water. Six microliters of 1 M DTT were added and samples were vortexed, then 11.5 5× sample buffer were added following by another vortex and boiling of samples prior to SDS-polyacrylamide gel electrophoresis and western blotting as described above.

### mRNA purification for m^6^A IP

Total RNA was extracted from cells harvested by vacuum filtration and flash frozen in liquid nitrogen using hot acid phenol as described above. mRNA was purified by 2 rounds of polyA selection as follows: 50 μl of SeraMag Oligo dT magnetic beads (GE Healthcare) were used per 100 μg total RNA. RNA was diluted to 1 μg/μl using RNase-free water (Life Technologies). Beads were washed twice in 2× RNA-binding buffer (NEB #E7492AA) and re-suspended in 100 μl 2× RNA-binding buffer. 100 μl total RNA were then added and samples were vortexed gently. Samples were then incubated at 65 °C for 5 min followed by 5 min at 4 °C. After gentle vortexing, samples were placed on a rotating rack for 15 min at 25 °C. Next, beads were placed on a magnetic rack and the supernatant was removed. Beads were washed 3 times in 200 μl Wash buffer (NEB #E7493AA). Next, the supernatant was discarded and beads were resuspended 50 μl Tris buffer (NEB #E7496A). Samples were incubated at 80 °C for 2 min, then at 25 °C for 5 min. Next, 50 μl of 2× RNA-binding buffer were added and the samples were gently vortexed and placed on the rotating rack again for 15 min. Next, samples were placed on the magnetic rack and the supernatant was removed. Beads were washed twice in 200 μl Wash buffer. The Wash buffer was then removed with the magnetic rack. To ensure complete removal of the Wash buffer, samples were then centrifuged briefly, placed back on the magnetic rack, and residual buffer was removed. mRNA was then eluted by addition of 10 μl Tris buffer, mixing by pipetting, and incubating at 80 °C for 2 min, then immediately placing the tubes on the magnetic rack. mRNA was transferred to a new tube and 1 μl was used for size distribution evaluation with Agilent Bioanalyzer to ensure that the mRNA is intact.

### m^6^A IP for m^6^A-Seq

m^6^A IP followed the procedure described in refs, ^21,23,70^ for three wild-type and three *ime4-cat* samples with some modifications as follows:

mRNA purified as described above from meiotic cells incubated for 5 h in SPO was fragmented using RNA Fragmentation Reagents (Life Technologies) by incubation at 70 °C for 2 min in a total volume of 10 μl. This resulted in fragments mostly around 70–120 nucleotides long as assessed using an Agilent Bioanalyzer. The volume was raised to 100 μl with RNase-free water and ethanol precipitated with 2 μl Glycogen Blue (Life Technologies).

Fragmented mRNA was re-suspended following ethanol precipitation in 13.5 μl RNase-free water. Then 0.5 μl Murine RNase inhibitor (NEB #M0314) was added, followed by 2 μl of 10× T4 polynucleotide kinase buffer (PKN, NEB #B0201), 1 μl T4 PKN enzyme (NEB #M0201), and 1 μl TURBO DNAse (Life Technologies). The total volume was 18 μl. Samples were then incubated at 37 °C for 30 min, and then 2 μl of 10 mM ATP were added and incubation resumed at 37 °C for another 30 min. This resulted in RNA fragments with a 5′-phosphate and a 3′-OH for subsequent library construction. The volume was then raised to 100 μl with RNase-free water and RNA was ethanol precipitated with 2 μl Glycogen Blue.

Following ethanol precipitation, fragmented, end-repaired mRNAs were re-suspended in 100 μl IPP buffer (150 mM NaCl, 0.1% IGEPAL CA-630 [Sigma], 10 mM Tris-HCl, pH 7.5). Ten microliters were set aside as input. Twenty-five microliters of Protein G magnetic beads (NEB) per sample were washed twice in IPP buffer and then resuspended in IPP buffer. Two microliters of anti-m^6^A antibody (Synaptic Systems #202 003) per sample were added to the beads and incubated on a rotating rack at room temperature for 30 min. Next, beads were washed twice in IPP buffer, re-suspended in 25 μl IPP buffer per sample, and 2 μl of RNase OUT (Life Technologies) per sample were added and the beads were kept on ice. Ninety microliters of fragmented mRNA were incubated at 70 °C for 5 min, then added to the beads and incubated at 4 °C for 2 h on a rotating rack. Next, beads were washed three times in IPP buffer, transferred to a new tube, and washed two more times. The beads were then centrifuged briefly and returned to the magnetic rack to remove residual IPP buffer. Antibody-bound RNA was then eluted using 30 μl of RLT buffer (Qiagen). Next, input and IP samples volumes were made up to 100 μl with RNase-free water, and RNA was ethanol precipitated with 2 μl Glycogen Blue.

Input and IP samples were re-suspended in 7 μl of RNase-free water. One microliter was used for determining RNA concentration with Agilent Bioanalyzer and the rest were used for library construction using NEBNext Multiplex Small RNA Library Prep Set of Illumina with primer set 2 (NEB #E7580S), according to the kit’s instructions. Adapters were diluted 1:3 and libraries were amplified with 15 PCR cycles. Libraries were run on an Agilent Bioanalyzer HS DNA chip, and using this data, the libraries were subsequently pooled so that the amounts of material between 160 and 270 bp were equal (ng). The final pooled library was size-selected on a PippinHT using a protocol set to elute from 140 to 280 bp, ensuring that fragments within the target of 160–270 bp were captured.

Following pooling of barcoded libraries, products were size selected to 140–270 bp using a Pippin prep and sequenced on an Illumina HiSeq generating 75 × 75 paired end reads.

### UTR extensions

All reads were quality controlled and adaptors removed using FASTX Toolkit (v 0.0.14) and cutadapt (v1.16). Since the Ensembl R64-1-1-80 (sacCer3) transcriptome annotations do not include UTRs, we computationally extended canonical gene 5′ and 3′ UTRs using RNA-Seq data from *S. cerevisiae*  SK288C 5 h meiotic cells. Using bowtie2 (v2.3.4.1), we mapped reads to the Ensembl R64-1-1-80 (sacCer3) genome in a strand-specific fashion and calculated per-base coverage for each annotated gene using the bedtools commands “bamtobed” and “genomeCoverageBed”^71^. The strand-specific per-base coverage was used to extend each UTR one base at a time until the coverage fell below 1/3 of the ORF’s median coverage, intersected an adjacent ORF, or reached 500 nt. UTR length was defined as the median length across all six input samples (three wild-type and three ime4-cat replicates).

### Read alignment

Reads were subsequently mapped against the Ensembl R64-1-1-80 (sacCer3) genome using Tophat2 (v2.1.1)^72^ with a custom GTF file of the transcriptome that including UTR extensions (as defined above). In addition, we used the options “–max-multihits 1–prefilter-multihits.” To connect each read pair, the Tophat2 output bam file was converted into a bedpe file with bedtools (v2.27) and this file was subsequently used to calculate per base coverages for each gene by using bedtools “genomeCoverageBed”^73^.

### Detection of m^6^A sites

Detection of m^6^A sites followed the procedure outlined in ref. ^23^. Briefly, putative m^6^A sites were identified using the following method: (1) Examination of the IP samples to identify m^6^A peaks within annotated genes including UTR extensions that were enriched compared to overall gene levels. (2) Comparison of IP to input enrichments (for wild-type and *ime4-cat* samples) to identify IP-specific peaks not present in inputs. (3) Comparison of wild-type and *ime4-cat* samples to identify WT-specific peaks not present in *ime4-cat*.

1. Peak detection: Genes with median expression >0 in all six IP samples were analyzed for potential m^6^A peaks. To identify peaks in the IP samples, each gene was scanned using sliding windows of 100 bases with a 50-base overlap. Each window was scored by calculating the mean coverage across the window divided by the median coverage of the gene. Windows having an enrichment score of >3 and a mean read depth of >10 were identified as peaks.
2. Identification of IP-specific peaks: The peak detection step was repeated for each input sample. Peaks present in IP and not in input were retained for all subsequent analyses.
3. Identification of wild-type-specific peaks: All peaks passing steps 1 and 2 in at least one sample were retained, and adjacent peaks were merged. For each merged peak, we recalculated the enrichment score as defined in step 1. m^6^A-dependent peaks were defined as those with a wild-type enrichment score divided by the *ime4-cat* enrichment score of at least 1.75, with the remaining peaks described as m^6^A independent. The summit of each peak was defined as the position with the highest coverage.

### Motif mapping and distance to the nearest motif

We calculated the distance from each of the 118 peak summits enriched in at least 2-fold in wild type compared to *ime4-cat* to the nearest RGAC motif on the same strand. As a control, we used 118 randomly selected summits from the m^6^A-independent peaks. As described in ref. ^23^, we identified 58 m^6^A-dependent summits within 5 nt of the nearest RGAC motif, extracted 24 nt centered on this motif, and used those as inputs for MEME (v5.0.0)^74^ to determine the consensus m^6^A motif.

### Metagene m^6^A-gene distribution

The position of each of the 118 m^6^A-dependent peak summits was expressed as a fraction of the corresponding gene’s transcript length. As a control, we performed the same analysis on the lowest scoring 118 m^6^A-independent peak summits.

### Gene ontology

GO categories analysis was carried using YeastMine (yeastmine.yeastgenome.org).

### m^6^A IP for RT-qPCR

Total RNA from meiotic cells incubated for 5 h in SPO was prepared as described above. One hundred micrograms of total RNA were spiked with 10 pg of in vitro transcribed Luciferase polyadenylated mRNA (Promega) for normalization, and mRNA was purified as described above and assayed using an Agilent Bioanalyzer. IP was done in the same way as for m^6^A-seq, except that intact rather than fragmented mRNA was used. cDNA from input and IP samples was generated with SuperScript III (Life Technonolgies) using 200 ng of mRNA. RT-pPCR was performed using SYBR green PCR master mix (Life Technologies) with primers listed in Supplementary Table 2 on an Applied Biosystem QuantStudio 5 instrument.

### Polysome profiling

All cultures were rapidly harvested by vacuum filtration and flash frozen in liquid nitrogen. Frozen pellets were mechanically lysed using a Sample Prep 6870 Freezer/Mill (Spex SamplePrep; 10 cycles of 2 min on, 2 min off at setting 10). Lysate powder was aliquoted and stored at −80 °C.

Crude lysates were prepared by re-suspending an aliquot of thawed lysate powder (approximately 800 μl of loosely packed powder, kept on ice for 3 min before re-suspension) in 1 ml of lysis buffer (10 mM Tris-HCl [pH 7.4], 5 mM MgCl_2_, 100 mM KCl, 1% Triton X-100, 1% Sodium Deoxycholate, 2 mM DTT, 0.4 mM cycloheximide, 20 U/ml SUPERase•In [Ambion], cOmplete EDTA-free Protease Inhibitor Cocktail [Roche]). The lysates were placed on a rotator mixer at 4 °C for 5 min to allow for re-suspension. Following brief vortexing, lysates were centrifuged at 1300 × *g* for 10 min, and 800 μl of the supernatant was loaded onto a 12.5 ml linear 10–50% (w/v) sucrose gradient (20 mM HEPES-KOH [pH 7.4], 5 mM MgCl_2_, 100 mM KCl, 2 mM DTT, 0.4 mM cycloheximide, 20 U/ml SUPERase•In). Gradients were centrifuged in a pre-chilled SW-41 Ti rotor at 222,000 × *g* *r*_max_ (acceleration mark “1,” deceleration mark “7”) for 2 h at 4 °C. Gradients were fractionated using a Piston Gradient Fractionator (Biocomp) in 1 ml fractions. *A*_254_ was monitored using an Econo UV Monitor (Biorad) and Gradient Profiler software (Biocomp, v2.07). Polysome fractions from each sample were pooled.

SDS (2%) with 40 μg/ml Proteinase K in RNase-free water was added 1:1 to pooled polysome or re-suspended input lysate in 15-ml conical tubes, followed by incubation for 30 min at 42 °C in an Eppendorf Thermomixer with shaking at 550 rpm. Then 2 ml of acid phenol were added and the samples were vortexed and incubated at 42 °C for another 5 min. Samples were then transferred to new tubes and spun down at 9400 × *g* for 10 min. The aqueous phases were transferred to new tubes and extracted with 2 ml chloroform followed by another vortex and centrifugation as before. The aqueous phases were moved to new tubes and RNA was precipitated by adding 200 μl sodium acetate, vortex, 4 μl Glycogen Blue (Life Technologies), vortex, and 2 ml isopropanol followed by overnight incubation at −20 °C. The next day, samples were centrifuged and RNA was re-suspended in 1 ml 70% ethanol in RNase-free water and moved to 1.5 ml microcentrifuge tubes, followed by 30 min centrifugation at 18,400 × *g* at 4 °C. RNA samples were re-suspended in RNase-free water.

mRNA from total RNA prior to gradient fractionation and from pooled polysomal fractions was purified as described above and used for library construction with the NEBNext Ultra RNA Library Prep Kit for Illumina (#E7530S), according to the manufacturer’s instructions.

### MazF mRNA restriction followed by PCR assay

Two hundred nanograms of polyA-selected mRNA from 5 h meiotic cells were denatured at 70 °C for 2 min and placed on ice. Next, 4 μl MazF buffer and 0.5 μl RNase inhibitor (NEB, M0314L) were added, and the volume was made up to 19 μl with RNase-free water. Next, 1 μl (20 U) of MazF (TaKaRa 2415A) were added and the samples were incubated at 37 °C for 2 h before ethanol precipitation with glycogen blue as described above. Next, RNA was resuspended in 8 μl RNAse-free water and cDNA was synthesized using SuperScript III with random hexamers (Life Technologies 18080–051) according to the manufacturer’s instructions. Two microliters of the resulting cDNA were used in subsequent PCR reactions using EmeraldAmp GT PCR master mix (TaKaRa RR310), and the products were run on a 2% agarose TBE gel stained with SYBER safe (Life Technologies S33102).

### Flow cytometric measurements of DNA replication

Five hundred microliters of meiotic cells were fixed in 1.5 ml 100% EtOH for at least 1 h in room temperature or overnight at 4 °C. Cells were then spun down briefly and re-suspended in 500 μl of 50 mM sodium citrate containing 40 μg/ml RNase A (Sigma), vortexed, and incubated at 50 °C for 1 h. Next, 10 μl of 20 μg/μl Proteinase K (Life Technologies) were added to each sample for another 1 h incubation at 50 °C followed by vortexing. Five hundred microliters of SYTOX green (Life Technologies) diluted 1:250 in 50 mM sodium citrate were then added to each sample. After vortexing, cells were transferred to 5 ml FACS tubes and analyzed using a BD CantoII instrument and BD FACS Diva software. Single cells were gated based on forward and side scattering, FITC-A, and FITC-W. Ten thousand events were counted per sample. Data were analyzed using the FlowJo 10 software.

### Meiotic division assay

Two hundred microliters of meiotic cells incubated for 24 h in SPO media were fixed in 500 μl 100% ethanol for at least 1 h at room temperature. Cells were centrifuged briefly and resuspended in 100 μl of 1 μg/ml DAPI in water and imaged using Nikon Eclipse Ti fluorescence microscope at ×100 magnification. Cells were counted and the percentage of cells with more than one nucleus was calculated.

### Sporulation assay

Two hundred microliters of meiotic cells incubated for 48 h in SPO media were fixed in 500 μl 100% ethanol for at least 1 h at room temperature. Cells were centrifuged briefly and resuspended in 100 μl water and imaged using Nikon Eclipse Ti fluorescence microscope at ×100 magnification. Cells were counted and the percentage of asci was calculated.

### Statistics and reproducibility

All statistical data were calculated using GraphPad Prism 7. Comparisons of data in Figs. 2c and 4f were performed using two-way analysis of variance. Fisher’s Exact Test was used for Supplementary Fig. 4d. For all other comparisons, two-tailed *t* tests were used. All experiments were repeated three times or performed in triplicates unless otherwise indicated.

### Reporting summary

Further information on research design is available in the Nature Research Reporting Summary linked to this article.

## Supplementary information

Supplementary Information

Peer Review

Reporting Summary

Description of Additional Supplementary Files

Supplementary Data 1

Supplementary Data 2

Source Data

## Data Availability

A reporting summary for this article is available as a Supplementary Information file. The source data underlying Figs. 1b–d, 2a–f, 3a–c, 4d, e, and 5b, d and Supplementary Fig. 1b–e are provided as a Source Data file. Sequencing data have been deposited in the NCBI Gene Expression Omnibus and are accessible through series accession number GSE130104. All data are available from the corresponding authors upon reasonable request.

## Code availability

The custom code used to detect m^6^A peaks in sequencing data is available to download from the Whitehead Institute bioinformatics core facility website at http://barc.wi.mit.edu/tools/
