# Supplementary material for: m6A modification of a 3′ UTR site reduces RME1 mRNA levels to promote meiosis
Source: Nat Commun. 2019 Jul 30;10:3414. doi: 10.1038/s41467-019-11232-7 (PMC6667471; doi:10.1038/s41467-019-11232-7)
Supplement: Supplementary file 1 — Supplementary Information [file 41467_2019_11232_MOESM1_ESM.pdf]

Supplementary Information for

m^{6}A modification of a 3′ UTR site reduces *RME1* mRNA levels to promote meiosis

Bushkin *et al*.

Supplementary Fig. 1

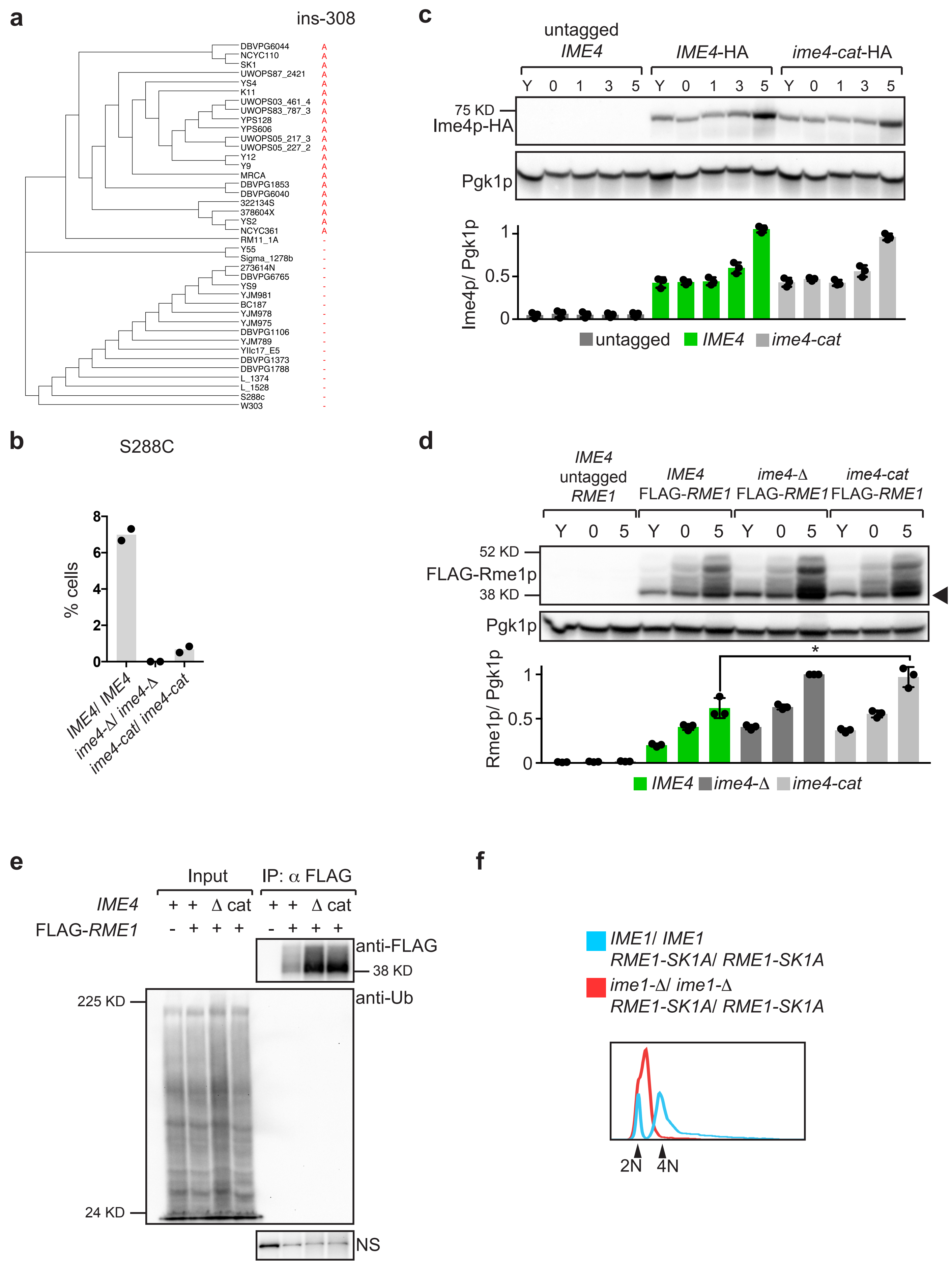

Supplementary Figure 1: *RME1* alleles in various strains. *IME4* is required for sporulation in S288C. Rme1p and Ime4p expression. *IME1* is required for DNA replication.

a) A phylogenetic tree of *RME1* ORF and 500 nucleotides upstream of it in various strains. The presence or absence of ins-308A is indicated in red. Most common lab strains, including S288C, W303, Sigma, and RM11, do not contain the insertion.

b) Meiotic nuclear divisions in *IME4*/ *IME4* and *ime4* mutants in the S288C strain as assayed by DAPI staining of nuclei after 24 hours in SPO medium. The percent of cells with two or more nuclei is indicated on the y-axis. Means and individual values from two experiments. At least 200 cells were counted per strain per experiment. Source data are provided in a Source Data file.

c) Western blot showing Ime4p and Ime4p-cat expression in exponential growth in YPD (Y), and during a time course in meiosis (0-5 hours). Pgk1p serves as loading control. A quantification of blots from three experiments is on the bottom with means, individual values, and s.d.. Source data are provided in a Source Data file.

d) Western blot showing Rme1p expression in indicated homozygous *IME4* backgrounds in logarithmic growth in YPD (Y) and meiosis (0 and 5 hours). An arrowhead marks the unmodified form of Rme1p. Pgk1p serves as loading control. A quantification of blots from three experiments is on the bottom with means, individual values, and s.d.. *p=0.019, two-tailed t-test. Source data are provided in a Source Data file.

e) Immunoprecipitation (IP) of tagged Rme1p from meiotic cells incubated in SPO media for 5 hours with an anti-FLAG antibody, followed by Western blotting with anti-FLAG and anti-Ubiquitin antibodies. Cell lysates before IP were used as controls for the anti-Ubiquitin antibody (Input). A non-specific anti-FLAG reactive band serves as loading control for the IP (NS). Source data are provided in a Source Data file.

f) Flow cytometry analysis of DNA content in *IME1*/ *IME1* and *ime1-Δ*/ *ime1-Δ* cells, both in the *RME1-SK1A*/ *RME1-SK1A* background. Cells were incubated for 24 hours in SPO media.

Supplementary Fig. 2

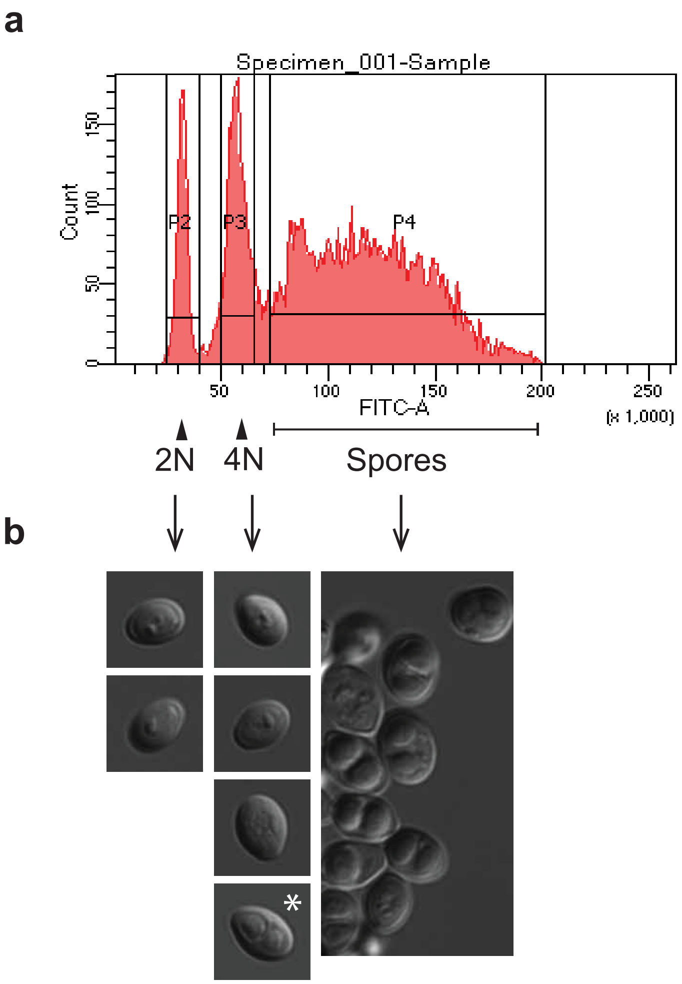

Supplementary Figure 2: Cell sorting of unsporulated and sporulated cells based on DNA content.
a) Flow cytometry analysis of DNA content in *IME4*/ *IME4* SK1 cells after 24 hours in SPO. Cells from 2N, 4N, and to the right of 4N (P2,P3, and P4, respectively), were sorted for microscopic examination.
b) Representative images of cells from the indicated fractions. Cells in P2 (2N) and P3 (4N) are unsporulated, while cells in P4 are asci and therefore 4N. Occasionally, a meiotic cell with pre-spore membranes can be observed (asterisk).

Supplementary Fig. 3

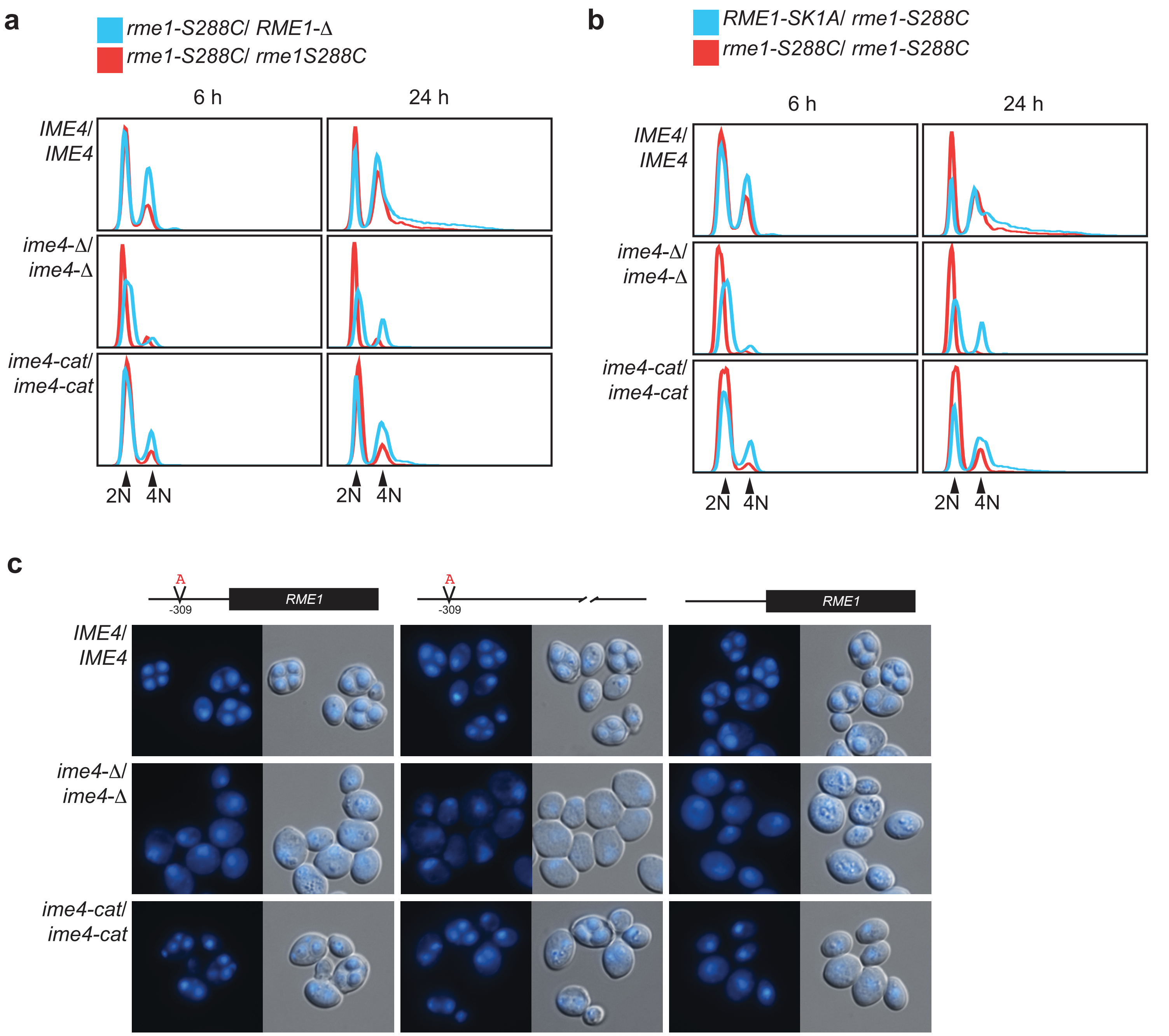

Supplementary Figure 3: *RME1* dosage tightly controls DNA replication. Meiotic divisions and sporulation in various *IME4* and *RME1* backgrounds.

a) Flow cytometry analysis of DNA content in *IME4*/ *IME4* and ime4 homozygous mutants (rows) in *rme1-S288C*/ *RME1-Δ* heterozygotes and *rme1-S288C* homozygotes over a meiotic time course (columns).

b) Flow cytometry analysis of DNA content in *IME4*/ *IME4* and *ime4* homozygous mutants (rows) in *RME1-SK1A*/ *rme1-S288C* heterozygotes and *rme1-S288C* homozygotes over a meiotic time course (columns).

c) Fluorescence (left panels) and light microscopy overlays (right panels) of DAPI stained *IME4*/ *IME4* and *ime4* homozygous mutants (rows) cells with the indicated *RME1* homozygous backgrounds (columns) after 24 hours in SPO medium.

Supplementary Fig. 4

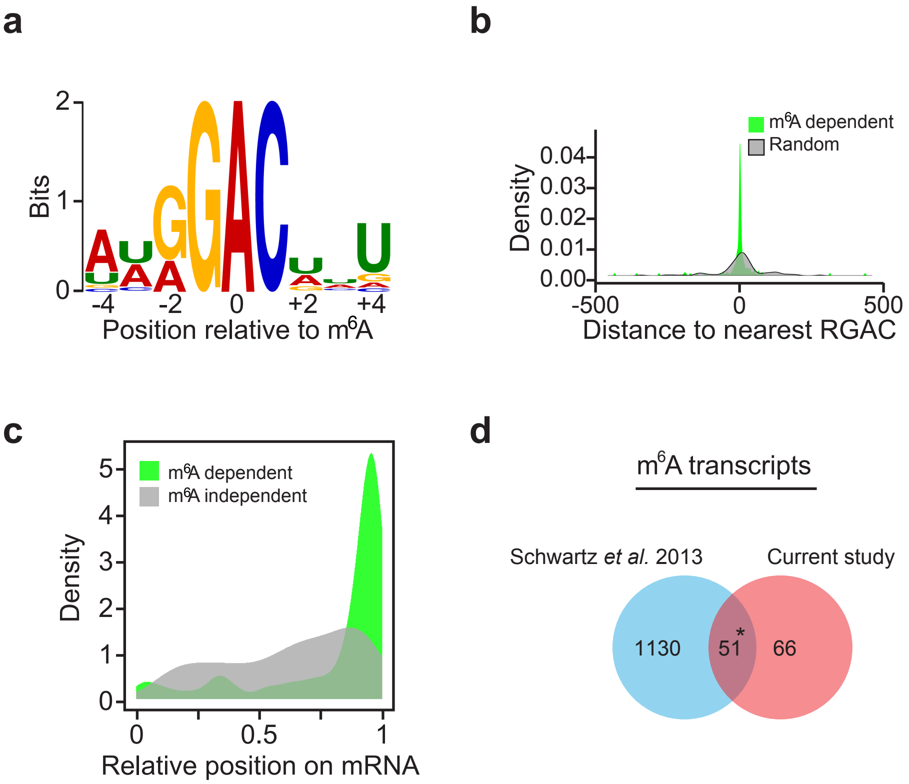

Supplementary Figure 4: m6A consensus motif and distribution. Methylated transcripts datasets overlap.
a) m6A methylation consensus motif identified using a MEME analysis of methylated sites.
b) Density plot of the distribution of distances between identified sites and the nearest RGAC motif in 118 sites enriched at least 2 in *IME4*/ *IME4* compared to *ime4-cat*/ *ime4-cat* (green) compared to 118 randomly selected sites (grey).
c) m6A methylation sites are concentrated in the 3′ ends of transcripts. The distributions of relative positions along a transcript (where 0 represents the 5′ end and 1 represents the 3′ end) of 118 m6A sites (green) and 118 m6A-independent sites (false-positives, grey).
d) Venn diagram showing the overlap between the m6A-seq data in SK1 23 and in SK288C *rme1-S288C*/ *rme1-S288C* (Current study). 51 mRNA are methylated in both data sets. *p<0.0001, Fisher's exact test.

Supplementary Fig. 5

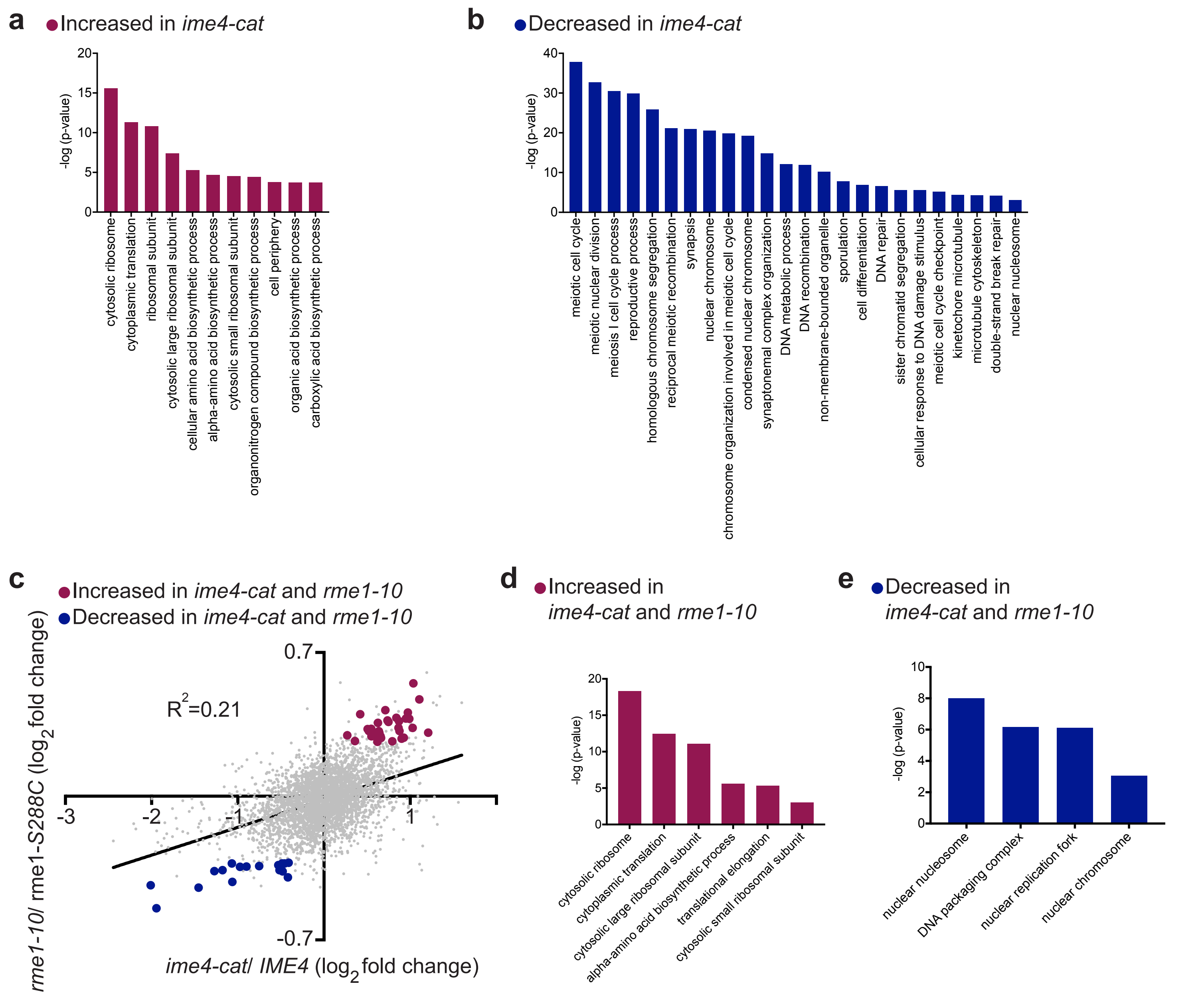

Supplementary Figure 5: GO categories of mRNAs enriched in *IME4* vs. *ime4-cat*, *ime4-cat* and *rme1-10* gene expression correlation and GO categories.

a) GO categories and p-values (Holm-Bonferroni) of mRNAs up-regulated in *ime4-cat*/ *ime4-cat* relative to *IME4*/ *IME4* cells from Fig. 5b.

b) GO categories and p-values (Holm-Bonferroni) of mRNAs up-regulated in *IME4*/ *IME4* relative to *ime4-cat*/ *ime4-cat* cells from Fig. 4b.

c) Correlation between mRNAs up-regulated (purple) and down-regulated (blue) with a p-value of less than 0.05 in a two-tailed t-test in *ime4-cat*/ *ime4-cat* and *rme1-10*/ *rme1-10* relative to their wild-type counterparts. Cells were incubated for 5 hours in SPO media. Data from 3 biological replicates.

d) GO categories and p-values (Holm-Bonferroni) of mRNAs up-regulated in Supplementary Figure 4c.

e) GO categories and p-values (Holm-Bonferroni) of mRNAs down-regulated in Supplementary Figure 4c.

Supplementary Table 1: Strains used in this study

Strain	Genotype	Background	Source
SAy821	*MAT* **a**/α *lys2/lys2 ho::LYS2/ho::LYS2*	SK1	Agarwala *et al*. 2012
FY2740	*MATa/MATα his3Δ1/his3Δ1 leu2Δ0/leu2Δ0 lys2Δ0/lys2Δ0 ura3Δ0/ura3Δ0*<br>*RME1(ins-308A)/RME1(ins-308A) TAO3(E1493Q)/TAO3(E1493Q) MKT1(D30G)/MKT1(D30G)*	SK288C<br>"WT"	Klomwieder, Winston 2011
GBy6	*MATa/MATα his3Δ1/his3Δ1 leu2Δ0/leu2Δ0 lys2Δ0/lys2Δ0 ura3Δ0/ura3Δ0*<br>*RME1(ins-308A)/RME1(ins-308A) TAO3(E1493Q)/TAO3(E1493Q) MKT1(D30G)/MKT1(D30G)*<br>*ime4-(D348A,W351A)/ime4-(D348A,W351A)*	SK288C	This study
BY4721 x BY4742	*MATa/MATα his3Δ1/his3Δ1 leu2Δ0/leu2Δ0 MET15/met15Δ0 Lys2/lys2Δ0 ura3Δ0/ura3Δ0*	S288C	Euroscaf collection
LD411	*MATa/MATα his3Δ1/his3Δ1 leu2Δ0/leu2Δ0 MET15/met15Δ0 Lys2/lys2Δ0 ura3Δ0/ura3Δ0*<br>*ime4::HIS4/ime4::HIS4*	S288C	This study
GBy30	*MATa/MATα his3Δ1/his3Δ1 leu2Δ0/leu2Δ0 MET15/met15Δ0 Lys2/lys2Δ0 ura3Δ0/ura3Δ0*<br>*ime4-(D348A,W351A)/ime4-(D348A,W351A)*	S288C	This study
GBy68	*MATa/MATα his3Δ1/his3Δ1 leu2Δ0/leu2Δ0 lys2Δ0/lys2Δ0 ura3Δ0/ura3Δ0*<br>*RME1-Δ,(ins-308A)/RME1-Δ,(ins-308A)*<br>*TAO3(E1493Q)/TAO3(E1493Q) MKT1(D30G)/MKT1(D30G)*	SK288C	This study
GBy71	*MATa/MATα his3Δ1/his3Δ1 leu2Δ0/leu2Δ0 lys2Δ0/lys2Δ0 ura3Δ0/ura3Δ0*<br>*RME1-Δ,(ins-308A)/RME1-Δ,(ins-308A)*<br>*TAO3(E1493Q)/TAO3(E1493Q) MKT1(D30G)/MKT1(D30G)*<br>*ime4-Δ/ime4-Δ*	SK288C	This study
GBy75	*MATa/MATα his3Δ1/his3Δ1 leu2Δ0/leu2Δ0 lys2Δ0/lys2Δ0 ura3Δ0/ura3Δ0*<br>*RME1-Δ,(ins-308A)/RME1-Δ,(ins-308A)*<br>*TAO3(E1493Q)/TAO3(E1493Q) MKT1(D30G)/MKT1(D30G)*<br>*ime4-(D348A,W351A)/ime4-(D348A,W351A)*	SK288C	This study
GBy146	*MATa/MATα his3Δ1/his3Δ1 leu2Δ0/leu2Δ0 lys2Δ0/lys2Δ0 ura3Δ0/ura3Δ0*<br>*RME1(ins-308A)/RME1(ins-308A) TAO3(E1493Q)/TAO3(E1493Q) MKT1(D30G)/MKT1(D30G)*<br>*ime4-Δ/ime4-Δ*	SK288C	This study
GBy222	*MATa/MATα his3Δ1/his3Δ1 leu2Δ0/leu2Δ0 lys2Δ0/lys2Δ0 ura3Δ0/ura3Δ0*<br>*TAO3(E1493Q)/TAO3(E1493Q) MKT1(D30G)/MKT1(D30G)*	SK288C	This study
GBy223	*MATa/MATα his3Δ1/his3Δ1 leu2Δ0/leu2Δ0 lys2Δ0/lys2Δ0 ura3Δ0/ura3Δ0*<br>*TAO3(E1493Q)/TAO3(E1493Q) MKT1(D30G)/MKT1(D30G)*<br>*ime4-Δ/ime4-Δ*	SK288C	This study
GBy225	*MATa/MATα his3Δ1/his3Δ1 leu2Δ0/leu2Δ0 lys2Δ0/lys2Δ0 ura3Δ0/ura3Δ0*	SK288C	This study

	TAO3(E1493Q)/TAO3(E1493Q) MKT1(D30G)/MKT1(D30G)<br>*ime4-(D348A,W351A)/ime4-(D348A,W351A)*		
GBy267	*MATa/MATα his3Δ1/his3Δ1 leu2Δ0/leu2Δ0 lys2Δ0/lys2Δ0*<br>*ura3Δ0/ura3Δ0*<br>*RME1(ins-308A)/RME1(ins-308A) TAO3(E1493Q)/TAO3(E1493Q)*<br>*MKT1(D30G)/MKT1(D30G)*<br>pGK21 [Shah and Clancy 1992]	SK288C	This study
GBy269	*MATa/MATα his3Δ1/his3Δ1 leu2Δ0/leu2Δ0 lys2Δ0/lys2Δ0*<br>*ura3Δ0/ura3Δ0*<br>*RME1(ins-308A)/RME1(ins-308A) TAO3(E1493Q)/TAO3(E1493Q)*<br>*MKT1(D30G)/MKT1(D30G)*<br>*ime4-Δ/ime4-Δ*<br>pGK21 [Shah and Clancy 1992]	SK288C	This study
GBy271	*MATa/MATα his3Δ1/his3Δ1 leu2Δ0/leu2Δ0 lys2Δ0/lys2Δ0*<br>*ura3Δ0/ura3Δ0*<br>*RME1(ins-308A)/RME1(ins-308A) TAO3(E1493Q)/TAO3(E1493Q)*<br>*MKT1(D30G)/MKT1(D30G)*<br>pGK21 [Shah and Clancy 1992]<br>*ime4-(D348A,W351A)/ime4-(D348A,W351A)*	SK288C	This study
GBy273	*MATa/MATα his3Δ1/his3Δ1 leu2Δ0/leu2Δ0 lys2Δ0/lys2Δ0*<br>*ura3Δ0/ura3Δ0*<br>*RME1(ins-308A)/RME1(ins-308A) TAO3(E1493Q)/TAO3(E1493Q)*<br>*MKT1(D30G)/MKT1(D30G)*<br>pGK21 [Shah and Clancy 1992]<br>*IME4/ime4-Δ*	SK288C	This study
GBy391	*MATa/MATα his3Δ1/his3Δ1 leu2Δ0/leu2Δ0 lys2Δ0/lys2Δ0*<br>*ura3Δ0/ura3Δ0*<br>*RME1(ins-308A)/RME1 TAO3(E1493Q)/TAO3(E1493Q)*<br>*MKT1(D30G)/MKT1(D30G)*	SK288C	This study
GBy393	*MATa/MATα his3Δ1/his3Δ1 leu2Δ0/leu2Δ0 lys2Δ0/lys2Δ0*<br>*ura3Δ0/ura3Δ0*<br>*RME1(ins-308A)/RME1 TAO3(E1493Q)/TAO3(E1493Q)*<br>*MKT1(D30G)/MKT1(D30G)*<br>*ime4-Δ/ime4-Δ*	SK288C	This study
GBy399	*MATa/MATα his3Δ1/his3Δ1 leu2Δ0/leu2Δ0 lys2Δ0/lys2Δ0*<br>*ura3Δ0/ura3Δ0*<br>*RME1(ins-308A)/RME1 TAO3(E1493Q)/TAO3(E1493Q)*<br>*MKT1(D30G)/MKT1(D30G)*<br>*ime4-(D348A,W351A)/ime4-(D348A,W351A)*	SK288C	This study
GBy413	*MATa/MATα his3Δ1/his3Δ1 leu2Δ0/leu2Δ0 lys2Δ0/lys2Δ0*<br>*ura3Δ0/ura3Δ0*<br>*RME1(ins-308A)/RME1(ins-308A) TAO3(E1493Q)/TAO3(E1493Q)*<br>*MKT1(D30G)/MKT1(D30G)*<br>*IME4/3xHA-IME4*	SK288C	This study
GBy415	*MATa/MATα his3Δ1/his3Δ1 leu2Δ0/leu2Δ0 lys2Δ0/lys2Δ0*<br>*ura3Δ0/ura3Δ0*<br>*RME1(ins-308A)/RME1(ins-308A) TAO3(E1493Q)/TAO3(E1493Q)*<br>*MKT1(D30G)/MKT1(D30G)*<br>*ime4-(D348A,W351A)/ime4-(D348A,W351A)-3xHA*	SK288C	This study
GBy432	*MATa/MATα his3Δ1/his3Δ1 leu2Δ0/leu2Δ0 lys2Δ0/lys2Δ0*	SK288C	This study

	ura3Δ0/ura3Δ0<br>*RME1(ins-308A)/RME1-Δ TAO3(E1493Q)/TAO3(E1493Q)*<br>*MKT1(D30G)/MKT1(D30G)*		
GBy433	*MATa/MATα his3Δ1/his3Δ1 leu2Δ0/leu2Δ0 lys2Δ0/lys2Δ0*<br>*ura3Δ0/ura3Δ0*<br>*RME1(ins-308A)/RME1-Δ TAO3(E1493Q)/TAO3(E1493Q)*<br>*MKT1(D30G)/MKT1(D30G)*<br>*ime4-Δ/ime4-Δ*	SK288C	This study
GBy435	*MATa/MATα his3Δ1/his3Δ1 leu2Δ0/leu2Δ0 lys2Δ0/lys2Δ0*<br>*ura3Δ0/ura3Δ0*<br>*RME1(ins-308A)/RME1-Δ TAO3(E1493Q)/TAO3(E1493Q)*<br>*MKT1(D30G)/MKT1(D30G)*<br>*ime4-(D348A,W351A)/ime4-(D348A,W351A)*	SK288C	This study
GBy470	*MATa/MATα his3Δ1/his3Δ1 leu2Δ0/leu2Δ0 lys2Δ0/lys2Δ0*<br>*ura3Δ0/ura3Δ0*<br>*RME1(ins-308A)/RME1(ins-308A) TAO3(E1493Q)/TAO3(E1493Q)*<br>*MKT1(D30G)/MKT1(D30G)*<br>*ime1-Δ/ime1-Δ*	SK288C	This study
GBy510	*MATa/MATα his3Δ1/his3Δ1 leu2Δ0/leu2Δ0 lys2Δ0/lys2Δ0*<br>*ura3Δ0/ura3Δ0*<br>*3xFLAG-RME1/3xFLAG-RME1 TAO3(E1493Q)/TAO3(E1493Q)*<br>*MKT1(D30G)/MKT1(D30G)*	SK288C	This study
GBy512	*MATa/MATα his3Δ1/his3Δ1 leu2Δ0/leu2Δ0 lys2Δ0/lys2Δ0*<br>*ura3Δ0/ura3Δ0*<br>*3xFLAG-RME1/3xFLAG-RME1 TAO3(E1493Q)/TAO3(E1493Q)*<br>*MKT1(D30G)/MKT1(D30G)*<br>*ime4-Δ/ime4-Δ*	SK288C	This study
GBy514	*MATa/MATα his3Δ1/his3Δ1 leu2Δ0/leu2Δ0 lys2Δ0/lys2Δ0*<br>*ura3Δ0/ura3Δ0*<br>*3xFLAG-RME1/3xFLAG-RME1 TAO3(E1493Q)/TAO3(E1493Q)*<br>*MKT1(D30G)/MKT1(D30G)*<br>*ime4-(D348A,W351A)/ime4-(D348A,W351A)*	SK288C	This study
GBy557	*MATa/MATα his3Δ1/his3Δ1 leu2Δ0/leu2Δ0 lys2Δ0/lys2Δ0*<br>*ura3Δ0/ura3Δ0*<br>*RME1-(A+129T)/RME1-(A+129T) TAO3(E1493Q)/TAO3(E1493Q)*<br>*MKT1(D30G)/MKT1(D30G)*	SK288C	This study

Supplementary Table 2: Primers used in this study

Oligo name	Sequence
ACT1 F	TTCCATCCAAGCCGTTTTGT
ACT1 R	CAGCGTAAATTGGAACGACGT
IME1 F	GACGTTGAAAAATCACCACCG
IME1 R	GAATTAAGAATAGGTTTTACTAAACTT
IME2 F	TTCCCCGGTGCTAACGAAA
IME2 R	TGGTGGAGCTGTGATGTGGTT
Luciferase F	CGCTGGAGAGCAACTGCATAA
Luciferase R	TTCCGCGTACGTGATGTTCAC
RME1 5'UTR F	GTGTCAACGCATTGGAACTGA
RME1 5'UTR R	GGCGATGGCACTGTTTTGT
RME1 ORF F	TGAGTTTGCCGCGCACTTA
RME1 ORF R	CAGTTGCTTGTTGGAAACCCA
RME1 stop F	GCGCAAAGACGCCTATAAGAG
RME1 stop R	ACAGTAGCTTTCATGCCTGCAC
RME1 m6A MazF F (P1)	GAAAACGCATGCAACTATGG
RME1 m6A MazF R (P2)	AGTAACCAACGTGGTGCCA
RME1 orf MazF F (P3)	AAGAATATTCGGCATCACCC
RME1 orf MazF R (P4)	CTAATGATAGTAGTAAACAGT

Supplementary Table 3: Models and predicted outcomes for single and double deletion mutant analysis

		Predicted DNA replication			
	***IME4*:**	**+/+**	Δ/Δ	**+/+**	Δ/Δ
**Model**	***RME1*:**	**+/+**	**+/+**	Δ/Δ	Δ/Δ
1	*RME1*→ *IME4*→ DNA	Yes	No	No	No
2	*RME1*→ *IME4* -I DNA	No	Yes	No	Yes
3	*RME1* -I *IME4*→ DNA	No	No	Yes	No
4	*RME1* -I *IME4* -I DNA	Yes	Yes	No	Yes
5	*IME4*→ *RME1*→ DNA	Yes	No	No	No
6	*IME4*→ *RME1* -I DNA	No	No	Yes	Yes
7	*IME4* -I *RME1*→ DNA	No	Yes	No	No
**8**	***IME4* -I *RME1* -I DNA**	**Yes**	**No**	**Yes**	**Yes**

Supplementary Table 3: Single and double deletion mutant analysis of DNA replication in *IME4* and *RME1*. Of the 8 possible models for the order of *IME4* and *RME1* in regulation of meiotic DNA replication, only model 8, in which *IME4* represses *RME1*, which represses DNA replication, is consistent with the observations.
