# Supplementary material for: m6A modification of a 3′ UTR site reduces RME1 mRNA levels to promote meiosis
Source: Nat Commun. 2019 Jul 30;10:3414. doi: 10.1038/s41467-019-11232-7 (PMC6667471; doi:10.1038/s41467-019-11232-7)
Supplement: Supplementary file 3 — Reporting Summary [file 41467_2019_11232_MOESM3_ESM.pdf]

nature research

Corresponding author(s): G. Guy Bushkin, Gerald R. Fink

Last updated by author(s): Jun 15, 2019

Reporting Summary

Nature Research wishes to improve the reproducibility of the work that we publish. This form provides structure for consistency and transparency in reporting. For further information on Nature Research policies, see Authors & Referees and the Editorial Policy Checklist.

Statistics

For all statistical analyses, confirm that the following items are present in the figure legend, table legend, main text, or Methods section.

n/a	Confirmed	
☐	☒	The exact sample size (*n*) for each experimental group/condition, given as a discrete number and unit of measurement
☐	☒	A statement on whether measurements were taken from distinct samples or whether the same sample was measured repeatedly
☐	☒	The statistical test(s) used AND whether they are one- or two-sided<br>*Only common tests should be described solely by name; describe more complex techniques in the Methods section.*
☐	☒	A description of all covariates tested
☐	☒	A description of any assumptions or corrections, such as tests of normality and adjustment for multiple comparisons
☐	☒	A full description of the statistical parameters including central tendency (e.g. means) or other basic estimates (e.g. regression coefficient) AND variation (e.g. standard deviation) or associated estimates of uncertainty (e.g. confidence intervals)
☐	☒	For null hypothesis testing, the test statistic (e.g. *F*, *t*, *r*) with confidence intervals, effect sizes, degrees of freedom and *P* value noted<br>*Give P values as exact values whenever suitable.*
☒	☐	For Bayesian analysis, information on the choice of priors and Markov chain Monte Carlo settings
☒	☐	For hierarchical and complex designs, identification of the appropriate level for tests and full reporting of outcomes
☐	☒	Estimates of effect sizes (e.g. Cohen's *d*, Pearson's *r*), indicating how they were calculated

Our web collection on statistics for biologists contains articles on many of the points above.

Software and code

Policy information about availability of computer code

Data collection	No software was written for this manuscript for the purpose of data collection.
Data analysis	1. For sequencing read quality control and adaptor trimming FASTX Toolkit (v 0.0.14) and cutadapt (v1.16) were used.<br>2. For extension of UTR sequences of the Ensembl R64-1-1-80 (sacCer3) transcriptome annotations bowtie2 (v2.3.4.1) and bedtools (v2.27) were used as described in the 'UTR extensions' section of the Methods.<br>3. For read alignment to the Ensembl R64-1-1-80 (sacCer3) transcriptome Tophat2 (v2.1.1) was used.<br>4. Per base coverages for each gene was calculated using bedtools (v2.27).<br>5. Detection of m6A sites followed the protocol described in Schwartz et al (23).<br>To detect peaks, a Perl script calling bedtools (v2.27) was written which applied a sliding window of 100 bases with a 50-base overlap to each gene. Subsequently, it calculated the mean coverage across the window and the median gene coverage which were used to calculate window fold change enrichment. Windows having an enrichment score of >3 and a mean read depth of >10 were identified as peaks. This script can be provided upon request.<br>6. The MEME (v5.0.0) software was used to determine the m6A consensus motif.<br>7. R (version 3.4.4) was used to produce metagene distribution and distance to nearest motif figures (Supplementary Fig. 4).

For manuscripts utilizing custom algorithms or software that are central to the research but not yet described in published literature, software must be made available to editors/reviewers. We strongly encourage code deposition in a community repository (e.g. GitHub). See the Nature Research guidelines for submitting code & software for further information.

Data

Policy information about availability of data

All manuscripts must include a data availability statement. This statement should provide the following information, where applicable:

- Accession codes, unique identifiers, or web links for publicly available datasets
- A list of figures that have associated raw data
- A description of any restrictions on data availability

The authors declare that the data supporting the findings of this study are available within the paper and its supplementary information files, and are available from the authors. The source data underlying Figs 1b–d, 2a–f, 3a–c, 4d,e, 5b,d and Supplementary Figs 1b–e are provided as a Source Data file. Sequencing data have been deposited into the NCBI Gene Expression Omnibus and are accessible through series accession number GSE130104.

Field-specific reporting

Please select the one below that is the best fit for your research. If you are not sure, read the appropriate sections before making your selection.

☒ Life sciences ☐ Behavioural & social sciences ☐ Ecological, evolutionary & environmental sciences

For a reference copy of the document with all sections, see nature.com/documents/nr-reporting-summary-flat.pdf

Life sciences study design

All studies must disclose on these points even when the disclosure is negative.

Sample size	We used three biological replicates for each experiment, or repeated the experiment three times, except where otherwise indicated, according to standards in the field.
Data exclusions	No data were excluded from analyses.
Replication	Fig. 1b,c: Three biological replicates.<br>Fig. 1d: Three independent experiments.<br>Fig. 2a-f: Three biological replicates.<br>Fig. 3a: Two to five independent experiments.<br>Fig. 3b,c: Three independent experiments.<br>Fig. 4a-e: Three biological replicates.<br>Fig. 5b-d: Three biological replicates.<br><br>All attempts at replication were successful.
Randomization	Replicates were allocated randomly within experimental groups.
Blinding	Investigators were blinded to experimental groups when analyzing RNA-seq data.

Reporting for specific materials, systems and methods

We require information from authors about some types of materials, experimental systems and methods used in many studies. Here, indicate whether each material, system or method listed is relevant to your study. If you are not sure if a list item applies to your research, read the appropriate section before selecting a response.

Materials & experimental systems

n/a	Involved in the study	
☐	☒	Antibodies
☒	☐	Eukaryotic cell lines
☒	☐	Palaeontology
☒	☐	Animals and other organisms
☒	☐	Human research participants
☒	☐	Clinical data

Methods

n/a	Involved in the study	
☒	☐	ChIP-seq
☐	☒	Flow cytometry
☒	☐	MRI-based neuroimaging

Antibodies

Antibodies used	M2 anti-FLAG-HRP (1:25,000, Sigma A8592-2MG), anti-Pgk1-HRP (1:500,000, Abcam 22C5D8), 10F3 anti-HA-HRP (1:10,000, Roche 12 013 819 001), P4D1 anti-Ub-HRP (1:10,000, Enzo BMLPW0935-0025), anti-m6A antibody (Synaptic Systems #202 003).
Validation	For epitope tags (FLAG and HA), antibodies were validated by the presence of a specific band at the anticipated molecular weight

in protein samples from tagged cells and absence of the band in untagged cells. Anti-PGK1 was validated by the presence of a specific band at the anticipated size, as described in the literature. Anti-Ub was validated by the appearance of the characteristic ladder as described in the literature. When using anti-m6A for m6A-seq, false positives were detected using control mRNAs from ime4 mutant cells.

Flow Cytometry

Plots

Confirm that:

- [ ] The axis labels state the marker and fluorochrome used (e.g. CD4-FITC).
- [ ] The axis scales are clearly visible. Include numbers along axes only for bottom left plot of group (a 'group' is an analysis of identical markers).
- [ ] All plots are contour plots with outliers or pseudocolor plots.
- [x] A numerical value for number of cells or percentage (with statistics) is provided.

Methodology

Sample preparation	500 ul of meiotic cells were fixed in 1.5 ml 100% EtOH for at least 1 hour in room temperature or overnight at 4°C. Cells were then spun down briefly and re-suspended in 500 ul of 50 mM sodium citrate containing 40 ug/ml RNase A (Sigma), vortexed, and incubated at 50oC for 1 hour. Next, 10 ul of 20 ug/ ul Proteinase K (Life Technologies) were added to each sample for another 1 hour incubation at 50°C followed by vortexing. 500 ul of SYTOX green (Life Technologies) diluted 1:250 in 50 mM sodium citrate were then added to each sample.
Instrument	BD CantoII
Software	BD FACS Diva, FlowJo 10
Cell population abundance	10,000 events were counted per sample.
Gating strategy	Single cells were gated on based on forward and side scattering, , FITC-A (20-250), and FITC-W (60-80).

- [x] Tick this box to confirm that a figure exemplifying the gating strategy is provided in the Supplementary Information.
