# Supplementary material for: m6A modification of a 3′ UTR site reduces RME1 mRNA levels to promote meiosis
Source: Nat Commun. 2019 Jul 30;10:3414. doi: 10.1038/s41467-019-11232-7 (PMC6667471; doi:10.1038/s41467-019-11232-7)
Supplement: Supplementary file 4 — Description of Additional Supplementary Files [file 41467_2019_11232_MOESM4_ESM.docx]

**Description of Additional Supplementary Files**

File Name: Supplementary Data 1

Description: m^6^A-seq data from meiotic cells in three biological replicates. IP enrichment values in *IME4* homozygotes and *ime4-cat* homozygotes. See Methods for details.

File Name: Supplementary Data 2

Description: RNA-seq data from meiotic cells. Mean normalized gene counts and p-values (two-tailed t-test) from three biological replicates in *ime4-cat* homozygotes vs. *IME4* homozygotes.
