# Supplementary material for: m6A modification of a 3′ UTR site reduces RME1 mRNA levels to promote meiosis
Source: Nat Commun. 2019 Jul 30;10:3414. doi: 10.1038/s41467-019-11232-7 (PMC6667471; doi:10.1038/s41467-019-11232-7)
Supplement: Supplementary file 2 — Peer Review [file 41467_2019_11232_MOESM2_ESM.pdf]

Reviewers' comments:

Reviewer #1 (Remarks to the Author):

This report focuses on the meiotic function of Ime4, a positive regulator of meiosis in budding yeast. Ime4 which is conserved in all eukaryotes functions as an N6-methyladenosine (m6A) on a consensus motif within mRNAs.
Yeast meiosis is characterized by a transcriptional cascade which can be roughly divided into early-middle, and late-meiosis-specific genes. Time of expression coordinate with time of function. For instance, the early genes encode for genes required for premeiotic DNA replication and meiotic recombination. IME1 encodes a transcriptional activator essential for the transcription of the early genes. The transcription of IME1 is regulated by nutrients as well as by the presence of The MATa and MATα alleles. The target of the MAT alleles is RME1, a DNA binding protein which represses the transcription of IME1. Ime4 is a positive regulator which is essential for the transcription of IME1. In this report the authors show that Ime4p acts upstream of Rme1p; it methylates RME1 mRNA. This methylation leads to destabilization of RME1 mRNA, it relieves Rme1p repression, and entry into meiosis. They identified the adenosine nucleotide in RME1 (A- 129 downstream of the stop codon) that depending on Ime4p, it is methylated. An A to T mutation of this residue led to an increase in the levels of RME1 mRNA and a significant decrease in DNA replication. This result strengthen their suggestion that methylation by Ime4p reduces the stability of Rme1p. Thus, there are two mechanisms that reduces the level of RME1 mRNA: repression of transcription by the Mata1p/Matα2p complex, and methylation by Ime4. Both mechanisms are essential for entry into meiosis.
The authors showed that in the absence of IME4, decreasing the level of Rme1p promoted premeiotic DNA replication, but cells arrested prior to nuclear division. On the other, in cells carrying the catalytic inactive mutation in IME4, nuclear division took place. These results suggest that Ime4p may have an additional function which is independent of its catalytic activity and is required for progression to MI. Finally, using m6A IP followed by RNA-seq they identified additional meiotic proteins that are subject this specific methylation. Their results reveal that similarly to Rme1p the consensus motif is present in the 3′-UTR.
This manuscript describes novel and interesting results which are properly controlled. Adenosine methylation seems to play a central role during development of many eukaryotes. In most cases their immediate effect is still obscure. This report shed a light on a specific mechanism, the effect of methylation in the 3′ UTR on the stability of the mRNA. Therefore, this report is suitable for publication in Nature, as it will be of interest to its broad spectrum of readers. I recommend acceptance of this paper with minor revisions as detailed below.

1. Title: "m6A modification of a 3′ UTR site represses RME1 to promote meiosis" The word represses is misleading, as it suggest post-translational effect, better, state your observation, for instance, destabilize RME1 RNA.
2. In the introduction or discussion, add the second mechanism that affects the level of RME1 RNA, transcriptional repression by the Mata1/Matα2 complex. Discuss why both mechanism are required.
3. Line 43 "Other members of the yeast protein complex include Slz1p and Mum2p".Be more precise are these 3 proteins the only members of the MIS complex?
4. Line 153 and Fig. 2E. At times 0, 1, and 3 hours in SPM (RME1-Δ/RME1-Δ strain) the level of IME1 RNA is lower in the ime4Δ strain in comparison to the IME4 strain. Please discuss this effect.
5. Line 168 "This role for Ime4p in sustaining high-level IME1 expression…" Possible explanations are required.
6. Fig. 3a: You examined DNA replication by examining the percentage of cells with 2N and 4N DNA content. The profile of cells at time 0 is an important control. Moreover, how did you calculate the percentage of cells with 4N? Is the increase in its level at 24 h took into account the shoulder of cells with "more than 4N"? It is possible that this shoulder represents clusters of unseparated cells, and therefore the quantification maybe incorrect.
7. Line 180 "Deletion of IME4 led to delayed meiotic DNA replication". In order to conclude "delayed replication", one need to see results at time 0; it is possible that there is no delay but differences of

profiles at this time.
8. Line 193 "meiotic DNA synthesis was measured and compared with that of the wild-type RME1 and IME4 strain and the respective single-deletion strains" where are the results?
9. Fig. 3b: Explain what you mean by "% cells with segregated DNA" do you means cells with more than 1 nuclei?
10. Line 229 "once as an m6A methyltransferase that drives DNA replication" The word drive DNA replication is misleading, leading the reader to think on a direct effect on DNA replication.
11. Fig. 3e: According to Shah and Clancy (1992) the transcription of IME4 is also induced by starvation. You may want to add it to the scheme.
12. Line 233 "To identify IME4 methylation targets critical for progression of the meiotic program" the rational for this experiment is not clear, as the additional function of Ime4p that is required for DNA replication, is independed on its catalytic activity.
13. Line 310 "This down-regulation is important because transcription of RME1 mRNA continues throughout meiosis" This statement is strengthened by the report that the expression of IME4 is induced under starvation.

Yona Kassir

Reviewer #2 (Remarks to the Author):

The manuscript of Bushkin et al addresses the role of mRNA methylation in yeast sporulation control. Their main conclusion is that a single methylation site in the 3' UTR of a single gene's transcripts (RME1) is responsible for (most) of the meiosis entry control by m6A. Highly synchronous meiotic cells are critical for these kinds of study, and even with the inclusion of the RME1-SK1A allele, synchronisation efficiency does not approach that of the SK1 wild type. Any inefficiencies in synchronisation are likely to amplify variation, particularly in the early stages of meiosis (contribution from G0 arrested, meiotic, premeiotic cells). A potential problem with synchrony is indicated by the mix of 2N and 4N cells (see Figure 3a left panel and compare with their similar data for synchronised SK1 cells - flow cytometry in Reference 10 Figure 5F).

Line 123-128 If Ime4-Delta and Ime4-cat 'do not have detectable methyl transferase activity' why does Fig 1c show only a ~1.4 fold decrease in m6A levels in these strains? Previous papers (eg ref 10 and earlier) have shown an absolute requirement for Ime4 for m6A formation. This rather small decrease is an important point that needs to be resolved –if it is background from rRNA species, then additional purification is required, if it is a genuine level of m6A in mRNA that is Ime4 independent, then this needs to be demonstrated.
The fold change in m6A should be stated.

Line 242 For Figure 4 it is necessary to show reads along the entire length of the RME1 transcript for input and IP (IME4 and ime4-cat), rather than just the small window. Reads per million should be included on the Y axis. This information is central to the argument being put forward and needs to be presented clearly. The fold enrichment could perhaps go into an additional panel or supplemental data.
The peak is quite broad, but the specific m6A site is not verified (this is important when the assumed suite is subsequently mutated). miCLIP or SCARLET could be used to confirm this A is m6A modified. In ref 22 (Schwartz et al), which includes some of the same authors, a thorough analysis of m6A sites at different time points was undertaken (in SK1). However, RME1 does not appear as methylated at any time point in these data sets. Are the authors suggesting that the upstream SNP (-308A) is influencing the downstream methylation? The transcript abundance shouldn't be affecting the MeRIPSeq outcome. The authors should address this discrepancy with their earlier data.

Line 267 (Fif 4f) The 1.46 fold expression change in the rme1-10 line based on RNA Seq should be verified by an orthogonal method to (eg qRT-PCR).

Other points
Line 75 Reference 6 Shah et al. does not say that IME4 is dispensable for meiosis.
From other references it is clear that IME4 is necessary for its proper timing and execution

Line 102 Reference here Ben-Ari et al. 2006 also. These authors found 4 additional genes (QTLs) linked to meiotic control. This indicates that a simple "reconstruction" of the SK1 strain in S288C is more complex than suggested in ref 52.

Line 105 "Quantitative PCR (RT-qPCR) showed that RME1-SK1A transcription is reduced nearly 4-fold…" – Strictly speaking, the RT-qPCR shows a reduction in transcript levels, this can only be attributed to transcription if decay rates are also measured or run on experiments performed.
Line 115 "Dispensable" for sporulation is perhaps a misleading phrase given the very low (and delayed) levels of sporulation seen in the SK1 strain when IME4 is deleted.

Line 132 Comment on why RME1 protein levels in the wildtype (extended Figure 1 d) rise so dramatically in the SPO medium compared to the YPD, when the lower vegetative levels of RME1 are presumably sufficient to suppress sporulation. If RME1 is a suppressor of sporulation, why is it so much higher at 5 hours when sporulation is taking place?

Line 145 For Figure 2 a and b it would be normal to show the rRNA profile from the fractions to confirm that there has not been any degradation. This is particularly relevant in meiosis where the resolution of different ribosome peaks is so low.

Lines 189 – 196 Perhaps refer to the model of Shah and Clancy (Fig 4 in ref 6) and discuss the differences or how the two models could be integrated.

Line 284 Explain the relevance of the sporulation experiments carried out at 37 C. This high temperature is inhibitory for meiosis (especially S288C cells which are temperature sensitive). Multiple pathways are likely affected, what is the specific relevance to meiosis and mRNA methylation?

Supplementary Fig 1 d Include the error bar for ime4 delta at 5 hours

Reviewer #3 (Remarks to the Author):

Bushkin et al. demonstrate m6A modification on the 3'UTR of RME1 regulates its expression and ultimately meiosis in yeast. Through genetic analyses of yeast strains with various RME1 and IME4 mutations, the authors propose a model of sequential repression. RNA-seq and m6A-seq data further support direct m6A modification as the key link. Overall, the experiments and interpretations are sound and logical. The effect of m6A at RME1 3´ UTR has exciting implications for understanding meiosis and RNA biology in general.

Major comments:
1. Examination of Rme1 protein levels is appropriate to substantiate the model. For example, quantification of RME1 (with the FLAG strain in 1d) protein should be provided for Fig 1b.
2. How m6A modification leads to destabilization and down-regulation of RME1 mRNA remains unclear. Some speculation is warranted. Presumably an m6A reader serves as an adaptor to the RNA decay machinery?

Minor comments:

1. The statement “Less progress has been made . . .” on page 3 is reads awkwardly.
2. A citation is needed for the statement on p. 4 “IME4 encodes a methyltransferase . . .”.
3. Sup Fig 1b is missing data to support the text “IME4 is dispensable for meiosis in SK1”.
4. Table 1 is a useful reference point, but perhaps Extended Data would be a better place.
5. For the m6A-seq experiment in Fig 4a/c, please offer some description and discussion on the other 33 high-confidence targets. Do the results match previous SK1 data?
6. It would be a bit nicer if the ratiometric comparisons in Figs 4a and b were the same (e.g. goth IME4/ime4); as-is, one is IME4/ime4 and the other is ime4/IME4.

Point-by-point response to referees' comments:

Reviewer 1:

1) Title: "m6A modification of a 3′ UTR site represses RME1 to promote meiosis" The word represses is misleading, as it suggest post-translational effect, better, state your observation, for instance, destabilize RME1 RNA.

The title is now: m^{6}A modification of a 3′ UTR site reduces *RME1* mRNA levels to promote meiosis.

2) In the introduction or discussion, add the second mechanism that affects the level of RME1 RNA, transcriptional repression by the Mata1/Matα2 complex. Discuss why both mechanism are required.

Inserted after line 72 (now 75): In diploids, *RME1* is repressed by the product of the mating type alleles, the a1/α2 complex (Covitz 1991). However, genetic and mutational analyses suggest that there are other unidentified mechanisms of *RME1* repression (Covitz 1991).

3) Line 43 "Other members of the yeast protein complex include Slz1p and Mum2p".Be more precise are these 3 proteins the only members of the MIS complex?

Line 43 (now 45): The text now reads: Other noncatalytic members of the yeast methyltransferase complex include Slz1p and Mum2p.

4) Line 163 and Fig. 2E. At times 0, 1, and 3 hours in SPM (RME1-Δ/RME1-Δ strain) the level of IME1 RNA is lower in the ime4Δ strain in comparison to the IME4 strain. Please discuss this effect.

The following text is added in line 175: We observed an increase in *IME1* mRNA levels in early time points in *IME4*/ *IME4* compared to *ime4-Δ*/ *ime4-Δ* cells in the *RME1-Δ*/ *RME1-Δ* background. This increase indicates that Ime4p has an additional role in *IME1* regulation independent of *RME1* (Fig. 2e), although this role was not further investigated. Perhaps Ime4p is involved in transduction of the nutritional starvation signals required to induce *IME1* (Kahana *et al*. 2010, Weidberg *et al.* 2016).

5) Line 168 "This role for Ime4p in sustaining high-level IME1 expression…" Possible explanations are required.

Line 173: The text now reads: This role of Ime4p in sustaining high-level *IME1* expression by overcoming Rme1p repression…

6) Fig. 3a: You examined DNA replication by examining the percentage of cells with 2N and 4N DNA content. The profile of cells at time 0 is an important control. Moreover, how did you calculate the percentage of cells with 4N? Is the increase in its level at 24 h took into account the shoulder of cells with "more than 4N"? It is possible that this shoulder represents clusters of unseparated cells, and

therefore the quantification maybe incorrect.

We added time 0 to Fig. 3a. Cells in the shoulder are asci that stain with SYTOX green upon spore wall formation. We added sorting data as a new Supplementary Figure S2. We added the following to line 193: Cell sorting followed by microscopy revealed that cells to the right of the 4N peak at 24 hours are asci (Supplementary Figure 2a, b). We added the following to the legend of Fig. 3a: % cells with 4N includes both sporulated and non-sporulated cells.

7) Line 180 “Deletion of IME4 led to delayed meiotic DNA replication”. In order to conclude “delayed replication”, one need to see results at time 0; it is possible that there is no delay but differences of profiles at this time.

See time 0 profiles added to Fig. 3a. There are no differences in 0 hours and the cells are synchronous.

8) Line 193 “meiotic DNA synthesis was measured and compared with that of the wild-type RME1 and IME4 strain and the respective single-deletion strains” where are the results?

Line 206: We added “Fig. 3a”.

9) Fig. 3b: Explain what you mean by “% cells with segregated DNA” do you means cells with more than 1 nuclei?

Fig. 3b y-axis now reads: % cells. We added the following to Fig. 3b legend: “The percent of cells with two or more nuclei is indicated on the horizontal axis.”

10) Line 229 “once as an m6A methyltransferase that drives DNA replication” The word drive DNA replication is misleading, leading the reader to think on a direct effect on DNA replication.

Line 243: We changed “drives” to “promotes”.

11) Fig. 3e: According to Shah and Clancy (1992) the transcription of IME4 is also induced by starvation. You may want to add it to the scheme.

While *IME1* is activated by starvation through carbon and nitrogen sensing elements in its promoter (Kahana *et al*. 2010), nutritional regulation of *IME4* is poorly understood. The model in Fig. 3e is simplified to focus on the positive regulation of *IME1* though the double negative action of *IME4* and *RME1*.

12) Line 233 “To identify IME4 methylation targets critical for progression of the meiotic program” the rational for this experiment is not clear, as the additional function of Ime4p that is required for DNA replication, is independed on its catalytic activity.

The rationale is that *ime4-cat/ ime4-cat cells* are defective in meiosis in the SK288C *rme1-S288C/ rme1-S288C* background, but not in SK1.

13) Line 310 “This down-regulation is important because transcription of RME1 mRNA continues throughout meiosis” This statement is strengthened by the report that the expression of IME4 is induced under starvation.

It would be interesting to explore the mechanism by which *IME4* is induced in starvation as a follow-up project.

Reviewer 2:

1) The manuscript of Bushkin et al addresses the role of mRNA methylation in yeast sporulation control. Their main conclusion is that a single methylation site in the 3’ UTR of a single gene’s transcripts (RME1) is responsible for (most) of the meiosis entry control by m6A. Highly synchronous meiotic cells are critical for these kinds of study, and even with the inclusion of the RME1-SK1A allele, synchronisation efficiency does not approach that of the SK1 wild type. Any inefficiencies in synchronisation are likely to amplify variation, particularly in the early stages of meiosis (contribution from G0 arrested, meiotic, premeiotic cells). A potential problem with synchrony is indicated by the mix of 2N and 4N cells (see Figure 3a left panel and compare with their similar data for synchronised SK1 cells - flow cytometry in Reference 10 Figure 5F).

Meiosis in *S. cerevisiae* is a part of the sporulation differentiation program, triggered in response to nutrient starvation. Specifically, yeast enter the program in the presence of a non-fermentable carbon source and the absence of a nitrogen source. SK1 is the most meiotic efficient lab strain. Typically more than 90% of cells enter meiosis and sporulate after 24 hours (Agarwala *et al.* 2012 for example). Because of its high efficiency SK1 is the main strain used in meiotic studies. For comparison, the canonical S288C strain only achieves <10% sporulation under the same conditions (Supplementary Figure 1b). The SK288C strain used in our study has a sporulation efficiency of ~50%, largely thanks to a single nucleotide insertion in the *RME1* promoter (Deutschbauer *et al.* 2005)

Many sporulation protocols, such as the one used in our study, include an overnight incubation in pre-sporulation media before switching to sporulation (SPO) media. The pre-sporulation media we used is BYTA, in which the carbon source is acetate (non-fermentable) but the cells are not starving for nitrogen. Thus, one of the two sporulation conditions is met, priming the cells for meiotic entry. The purpose of the pre-sporulation step is to achieve synchronicity, so that cells will progress through the different steps in meiosis and sporulation together once transferred to SPO. In our experience with dozens of sporulation experiments, SK288C is synchronous while using our protocol: The cells that replicate their DNA do so in synchrony, while the rest will not enter meiosis even after 24 hours. We’ve added the 0 time point to figure 3a to better illustrate the synchronicity of our meiotic cultures.

2) Line 123-128 If Ime4-Delta and Ime4-cat 'do not have detectable methyl transferase activity' why does Fig 1c show only a ~1.4 fold decrease in m6A levels in these strains? Previous papers (eg ref 10 and earlier) have shown an absolute requirement for Ime4 for m6A formation. This rather small decrease is an important point that needs to be resolved –if it is background from rRNA species, then additional purification is required, if it is a genuine level of m6A in mRNA that is Ime4 independent, then this needs to be demonstrated.

The fold change in m6A should be stated.

Line 123-128: The m^{6}A detected in *ime4* mutants likely comes from non-mRNA species present in the sample, despite two rounds of polyA selection. These include the product of the *LSR1* gene, U2 splicosomal RNA, which is known to have m^{6}A (Bringmann and Luhrmann 1986) and is one of the most transcribed genes in yeast, present at levels orders of magnitudes higher than any mRNA of similar size (1175 nt, Ares 1986). Other potential high abundance sources of m^{6}A are tRNAs. In support of the presence of snRNA and tRNA species that persist after two rounds of polyA selection our mass-spec analysis detected 1-methyladenosine and 2'-O-metyladenosine.

Regardless of the source of excess m^{6}A, the main point of this experiment was to test whether the *ime4-cat*/ *ime4-cat* mutant lost its m^{6}A catalytic activity. The answer is yes, as evident by the fact *ime4-cat*/ *ime4-cat* cells have the same amount of m^{6}A as *ime4-Δ*/ *ime4-Δ*. The *ime4-cat* mutant carries two alanine substitutions of conserved aspartic acid and tryptophan residues in the predicted catalytic site (Clancy *et al.* 2002), and has been used extensively in yeast and in some works in mammals (For example Schwartz *et al*. 2013 for yeast, and Choe *et al.* 2018 for mammals). However, to date there have been no demonstration that it is indeed catalytically inactive and expressed to the same level as the WT allele. We address these two gaps in the literature in Fig. 1c and Supplementary Figure 1c. It is important to note that the conclusions in our paper do not rely on complete loss of m^{6}A activity in *ime4-cat*/ *ime4-cat*, because there are clear cellular and molecular phenotypes for this mutant, which are not a result of reduced Ime4-cat protein expression.

3) Line 242 For Figure 4 it is necessary to show reads along the entire length of the RME1 transcript for input and IP (IME4 and ime4-cat), rather than just the small window. Reads per million should be included on the Y axis. This information is central to the argument being put forward and needs to be presented clearly. The fold enrichment could perhaps go into an additional panel or supplemental data. The peak is quite broad, but the specific m6A site is not verified (this is important when the assumed suite is subsequently mutated). miCLIP or SCARLET could be used to confirm this A is m6A modified.
   In ref 22 (Schwartz et al), which includes some of the same authors, a thorough analysis of m6A sites at different time points was undertaken (in SK1). However, RME1 does not appear as methylated at any time point in these data sets. Are the authors suggesting that the upstream SNP (-308A) is influencing the downstream methylation? The transcript abundance shouldn't be affecting the MeRIPSeq outcome. The authors should address this discrepancy with their earlier data.

Fig. 4d now shows reads for the entire *RME1* transcript. Moreover, we Confirmed the 3′ UTR methylation site using an orthogonal method to complement our m^{6}A -seq result. The method relies on MazF, an RNA restriction enzyme that cleaves RNA at ACA sequences, but not in m^{6}ACA sequences (Imanishi *et al.* 2017). This analysis is in Fig. 4e. We added the following text to describe the new analysis (line 282): "In order to probe the methylation status of +129A, we used MazF, a methylation-sensitive RNA restriction enzyme (Imanishi *et al.* 2017), in a recently described PCR-based assay (Garcia-Campos *et al.* 2019). MazF cleaves RNA at ACA sites, but not at m^{6}ACA sites. The ACA sequence is part of the GGACA sequence flanking the +129A (Fig. 4e). We digested purified mRNA from *IME4*/ *IME4* and *ime4-cat*/ *ime4-cat* cells with MazF, and

then reverse transcribed with random hexamers. PCR amplification of the resulting cDNA using primers that flank +129A in the *RME1* 3′ UTR yielded a product in cDNA prepared from *IME4*/ *IME4*, but not from *ime4-cat*/ *ime4-cat* (Fig. 4e). Thus, +129A in the *RME1* 3′ UTR is protected from cleavage by MazF in mRNA derived from cells with a functional methyltransferase. This result confirms that the *RME1* 3′ UTR from *IME4*/ *IME4* cells is methylated at the +129A position in *IME4*/ *IME4* cells but not in *ime4-cat*/ *ime4-cat* cells."

The m^6A -seq analysis performed in (Schwartz *et al*. 2013) was done in the SK1 strain that carries the *RME1-SK1A* allele. *RME1-SK1A* expression is greatly reduced compared with the *rme1-S288C* allele due to a promoter insertion (Fig.1a and 1b). It is possible that the *RME1* m^6A site was missed because of this reduced expression. Of note, *ime4-cat*/ *ime4-cat* cells in the SK1 background complete meiosis and sporulation, whereas they do not in SK288C carrying the rme1-S288C allele. Therefore, our analysis was performed in a genetic background in which the methyltransferase activity of *IME4* is essential for meiosis, unlike in SK1. In order to compare our results with previous data, we added a Venn diagram of methylated genes in SK1 vs. SK288C in Supplementary Figure 4d. We added the following text (line 256): "Of the 117 methylated mRNAs we identified, 51 were also identified in the previous m^6A-seq dataset from the meiotic-efficient SK1 (Supplementary Figure 4d, $p < 0.0001$, Fisher's Exact Test (Schwartz *et. al* 2013)),."

4) Line 267 (Fif 4f) The 1.46 fold expression change in the rme1-10 line based on RNA Seq should be verified by an orthogonal method to (eg qRT-PCR).

This value is derived from triplicate RNA-seq measurements and has an associated p-value showing 1.46-fold is a significant difference. We used RNA-seq since it is more sensitive and more reliable than Western blotting or RT-qPCR, in part because RNA-seq detects differences relative to the entire transcriptome rather than to a limited set of arbitrarily chosen mRNAs.

5) Line 75 Reference 6 Shah et al. does not say that IME4 is dispensable for meiosis.
   From other references it is clear that IME4 is necessary for its proper timing and execution

IME4 is necessary for meiosis in the strain where it was first discovered (Shah and Clancy 1992), but not in SK1 (Agarwala *et al.* 2012). The two references were at the end of the sentence, which may have confused the referee. We separated the references (line 80).

6) Line 102 Reference here Ben-Ari et al. 2006 also. These authors found 4 additional genes (QTLs) linked to meiotic control. This indicates that a simple "reconstruction" of the SK1 strain in S288C is more complex than suggested in ref 52.

We changed "three" to "several" (line 102) and added the Ben-Ari *et al*. 2006 reference at the end of the sentence.

7) Line 105 "Quantitative PCR (RT-qPCR) showed that RME1-SK1A transcription is reduced nearly 4-fold…" – Strictly speaking, the RT-qPCR shows a reduction in

transcript levels, this can only be attributed to transcription if decay rates are also measured or run on experiments performed.

We changed “transcription” to “expression” (line 110).

8) Line 115 “Dispensable” for sporulation is perhaps a misleading phrase given the very low (and delayed) levels of sporulation seen in the SK1 strain when IME4 is deleted.

In (Agarwala *et al.* 2012), SK1 *ime4-Δ*/ *ime4-Δ* cells sporulate to 50% after 24 hours. This is in sharp contrast to 0% in S288C and in SK288C under the same conditions, and shows that *IME4* is dispensable for sporulation in SK1.

9) Line 132 Comment on why RME1 protein levels in the wildtype (extended Figure 1 d) rise so dramatically in the SPO medium compared to the YPD, when the lower vegetative levels of RME1 are presumably sufficient to suppress sporulation. If RME1 is a suppressor of sporulation, why is it so much higher at 5 hours when sporulation is taking place?

Others (Primig *et al.* 2000) and we have noticed that *RME1* expression increases during meiosis, despite its role as a repressor of meiosis. The reason for this increase is unclear, but it suggests that *RME1* levels must be down-regulated post-transcriptionally.

10) Line 145 For Figure 2 a and b it would be normal to show the rRNA profile from the fractions to confirm that there has not been any degradation. This is particularly relevant in meiosis where the resolution of different ribosome peaks is so low.

The monosome peak is easily distinguishable from the polysomes, which are the fractions used for the analysis. Cells were harvested by vacuum filtration and frozen in liquid nitrogen, then milled in a cryo-miller prior to RNA extraction with RNAse inhibitors at 4oC. There was no obvious RNA degradation under these conditions. RNA degradation would have resulted in loss of monosome peaks.

11) Lines 189 – 196 Perhaps refer to the model of Shah and Clancy (Fig 4 in ref 6) and discuss the differences or how the two models could be integrated.

We added (line 209): “*IME4* is known to be required for expression of *IME1* (Shah and Clancy 1992). Our results show that this positive genetic relationship is achieved via negative regulation of *RME1* (Fig. 3e).”

12) Line 284 Explain the relevance of the sporulation experiments carried out at 37 C. This high temperature is inhibitory for meiosis (especially S288C cells which are temperature sensitive). Multiple pathways are likely affected, what is the specific relevance to meiosis and mRNA methylation?

We added (line 313): “a restrictive temperature that decreases meiotic efficiency…”.

13) Supplementary Fig 1 d Include the error bar for ime4 delta at 5 hours

Supplementary Figure 1d: There is no error bar for *ime4-Δ/ ime4-Δ* because the data is from three separate Western blots where signals from Rme1p bands were normalized relative to the highest intensity band, which was assigned a value of 1. The highest Rme1p intensity band in all 3 blots was in *ime4-Δ/ ime4-Δ*.

Reviewer 3:

Major comments

1) Examination of Rme1 protein levels is appropriate to substantiate the model. For example, quantification of RME1 (with the FLAG strain in 1d) protein should be provided for Fig 1b.

Figure 1b shows that *RME1* expression in the same genetic background can differ by ~5 fold based on the presence of a single promoter insertion. With regards to our model, we tested Rme1p levels in three different *IME4* backgrounds in Fig. 1e and Supplementary Figures 1d and 1e, all clearly and reproducibly showing that Rme1p is down regulated by Ime4p m^6A activity.

2) How m6A modification leads to destabilization and down-regulation of RME1 mRNA remains unclear. Some speculation is warranted. Presumably an m6A reader serves as an adaptor to the RNA decay machinery?

We added (line 370): "The increase of *RME1* mRNA levels in *IME4/ IME4 rme1-10/ rme1-10* cells suggests a destabilizing effect for m^6A on *RME1* transcripts. This is consistent with a role for m^6A in mRNA destabilization in mammals mediated by the reader protein YTHDF2 (Wang *et al.* 2014), which has a homolog in yeast, *MRB1* (Schwartz *et al*. 2013)."

Minor comments:

1) The statement "Less progress has been made . . ." on page 3 is reads awkwardly.

We rephrased to (line 84): Less progress has been made in distinguishing the consequential m^6A sites out of the entire m^6A methylome.

2) A citation is needed for the statement on p. 4 "IME4 encodes a methyltransferase . . .".

We added reference 4 (Clancy *et al*. 2002).

3) Sup Fig 1b is missing data to support the text "IME4 is dispensable for meiosis in SK1".

The data are in the provided reference 10 (Agarwala *et al.* 2012).

4) Table 1 is a useful reference point, but perhaps Extended Data would be a better place.

Table 1 is now Supplementary Table 3, and Supplementary tables 3 and 4 are now 4 and 5, respectively.

5) For the m6A-seq experiment in Fig 4a/c, please offer some description and discussion on the other 33 high-confidence targets. Do the results match previous SK1 data?

We added a comparison of methylated genes in SK1 vs. SK288C in Supplementary Figure 4d, and modified the text as noted above.

6) It would be a bit nicer if the ratiometric comparisons in Figs 4a and b were the same (e.g. goth IME4/ime4); as-is, one is IME4/ime4 and the other is ime4/IME4.

For aesthetic reasons, we prefer to keep the axes in Figures 4a and 4b as they are in order to have *RME1* on the same side in both panels.

REVIEWERS' COMMENTS:

Reviewer #1 (Remarks to the Author):

Review of the revised manuscript by Bushkin et al.
I read the reply of the authors to my comments and they properly addressed all my comments. I also read their responses to reviewers 2 and 3 comments, and was satisfied by their answers and corrections made in the manuscript. I reread the revised manuscript and find it suitable for publication. My recommendation: this manuscript should be accepted for publication.

Reviewer #2 (Remarks to the Author):

2) I appreciate that purification of good quality mRNA is technically difficult. However, other researchers in the field regularly carry out m6A measurements without incurring this level of contamination from non-mRNA species. I am surprised by the response that this contamination with other sources of m6A doesn't matter because the ime4-Δ/ ime4-Δ and the ime4-cat/ ime4-cat samples show similar m6A levels, and so presumably have the same level of contamination. Whilst technically demanding, the required experiments are not time consuming, and having confidence in the reported values should be a minimum requirement. This is especially true when the publication is in a high impact journal that is likely to shape the research work of other groups.

10) "… There was no obvious RNA degradation under these conditions. RNA degradation would have resulted in loss of monosome peaks."
Could the authors state how lack of degradation was assessed?
The statement that RNA degradation will result in loss of monosomes is not true. Treatment with ribonuclease followed by 80S purification is a standard protocol used for ribosome footprinting.

Reviewer #2 (Remarks to the Author):

2) I appreciate that purification of good quality mRNA is technically difficult. However, other researchers in the field regularly carry out m6A measurements without incurring this level of contamination from non-mRNA species. I am surprised by the response that this contamination with other sources of m6A doesn’t matter because the ime4-Δ/ ime4-Δ and the ime4-cat/ ime4-cat samples show similar m6A levels, and so presumably have the same level of contamination. Whilst technically demanding, the required experiments are not time consuming, and having confidence in the reported values should be a minimum requirement. This is especially true when the publication is in a high impact journal that is likely to shape the research work of other groups.

Since the reviewer is concerned about these data, and since they are not necessary to support our overall conclusion, we’ve opted to remove panel 1c and all references to it.

10) “… There was no obvious RNA degradation under these conditions. RNA degradation would have resulted in loss of monosome peaks.”
Could the authors state how lack of degradation was assessed?
The statement that RNA degradation will result in loss of monosomes is not true. Treatment with ribonuclease followed by 80S purification is a standard protocol used for ribosome footprinting.

We meant to say polysome peaks. We only used polysomes. RNA degradation would have resulted in loss of the polysome peaks.
